# Deepfake Media Forensics: Status and Future Challenges

**DOI:** 10.3390/jimaging11030073

**Published:** 2025-02-28

**Authors:** Irene Amerini, Mauro Barni, Sebastiano Battiato, Paolo Bestagini, Giulia Boato, Vittoria Bruni, Roberto Caldelli, Francesco De Natale, Rocco De Nicola, Luca Guarnera, Sara Mandelli, Taiba Majid, Gian Luca Marcialis, Marco Micheletto, Andrea Montibeller, Giulia Orrù, Alessandro Ortis, Pericle Perazzo, Giovanni Puglisi, Nischay Purnekar, Davide Salvi, Stefano Tubaro, Massimo Villari, Domenico Vitulano

**Affiliations:** 1 Department of Computer, Control and Management Engineering, Sapienza University of Rome, 00185 Roma, Italy; amerini@diag.uniroma1.it (I.A.); majid@diag.uniroma1.it (T.M.); 2 Department of Information Engineering and Mathematics, University of Siena, 53100 Siena, Italy; mauro.barni@unisi.it (M.B.); nischay.purnekar@student.unisi.it (N.P.); 3 Department of Mathematics and Computer Science, University of Catania, 95125 Catania, Italy; sebastiano.battiato@unict.it (S.B.); alessandro.ortis@unict.it (A.O.); 4 Department of Electronics, Information and Bioengineering (DEIB), Politecnico di Milano, 20133 Milano, Italy; paolo.bestagini@polimi.it (P.B.); sara.mandelli@polimi.it (S.M.); davide.salvi@polimi.it (D.S.); stefano.tubaro@polimi.it (S.T.); 5 Department of Information Engineering and Computer Science, University of Trento, 38123 Trento, Italy; giulia.boato@unitn.it (G.B.); francesco.denatale@unitn.it (F.D.N.); andrea.montibeller@unitn.it (A.M.); 6 Truebees S.r.l., 20900 Monza, Italy; 7 Department of Basic and Applied Sciences for Engineering, Sapienza University of Rome, 00185 Roma, Italy; vittoria.bruni@uniroma1.it (V.B.); domenico.vitulano@uniroma1.it (D.V.); 8 CNIT, National Inter-University Consortium for Telecommunications, 50134 Florence, Italy; roberto.caldelli@cnit.it; 9 Department of Engineering and Sciences, Universitas Mercatorum, 00186 Rome, Italy; 10 CNIT, University of Trento, 38122 Trento, Italy; 11 IMT School for Advanced Studies, 55100 Lucca, Italy; rocco.denicola@imtlucca.it; 12 Department of Electrical and Electronic Engineering, University of Cagliari, 09123 Cagliari, Italy; marcialis@unica.it (G.L.M.); marco.micheletto@unica.it (M.M.); giulia.orru@unica.it (G.O.); 13 Department of Information Engineering, University of Pisa, 56122 Pisa, Italy; pericle.perazzo@unipi.it; 14 Department of Mathematics and Computer Science, University of Cagliari, 09124 Cagliari, Italy; puglisi@unica.it; 15 MIFT Department, University of Messina, Viale F. Stagno d’Alcontres, 31, 98166 Messina, Italy; massimo.villari@unime.it

**Keywords:** media forensics, deepfake detection, deepfake attribution and recognition, deepfake authentication techniques, audio deepfake detection

## Abstract

The rise of AI-generated synthetic media, or deepfakes, has introduced unprecedented opportunities and challenges across various fields, including entertainment, cybersecurity, and digital communication. Using advanced frameworks such as Generative Adversarial Networks (GANs) and Diffusion Models (DMs), deepfakes are capable of producing highly realistic yet fabricated content, while these advancements enable creative and innovative applications, they also pose severe ethical, social, and security risks due to their potential misuse. The proliferation of deepfakes has triggered phenomena like “Impostor Bias”, a growing skepticism toward the authenticity of multimedia content, further complicating trust in digital interactions. This paper is mainly based on the description of a research project called FF4ALL (FF4ALL-Detection of Deep Fake Media and Life-Long Media Authentication) for the detection and authentication of deepfakes, focusing on areas such as forensic attribution, passive and active authentication, and detection in real-world scenarios. By exploring both the strengths and limitations of current methodologies, we highlight critical research gaps and propose directions for future advancements to ensure media integrity and trustworthiness in an era increasingly dominated by synthetic media.

## 1. Introduction

The rapid advancement of artificial intelligence (AI) has given rise to a new wave of synthetic media, widely known as deepfakes. These are highly realistic images, audio, and videos generated using sophisticated AI techniques such as Generative Adversarial Networks (GANs) [1] and Diffusion Models (DMs) [2]. While offering unprecedented creative possibilities, these technologies have also raised substantial ethical and security concerns, posing risks in domains such as entertainment, politics, and cybersecurity. Deepfakes are often indistinguishable from authentic media, which has led to their misuse in spreading misinformation, impersonation, and other malicious activities. Recent advancements in deepfake technology have enabled highly realistic synthetic media, leading to a surge in fraud cases. A notable example occurred in 2024, when a deepfake video of Elon Musk (https://www.nytimes.com/interactive/2024/08/14/technology/elon-musk-ai-deepfake-scam.html, last accessed on 17 February 2025) was used to promote a cryptocurrency scam, convincing an 82-year-old retiree to invest 690,000, ultimately resulting in a complete financial loss. In 2024, AI technologies were also used for the cyber fraud. In a recent example, the British engineering firm Arup fell victim to a deepfake scam (https://edition.cnn.com/2024/02/04/asia/deepfake-cfo-scam-hong-kong-intl-hnk/index.html, last accessed on 17 February 2025), where attackers impersonated the company’s Chief Financial Officer and other employees during a video conference. A staff member was deceived into authorizing 15 transactions totaling approximately 25.6 million. Unfortunately, deepfake technology has also introduced new threats to democratic processes. In January 2024, during the New Hampshire Democratic primary, an AI-generated robocall impersonating President Joe Biden (https://www.theguardian.com/us-news/article/2024/may/23/biden-robocall-indicted-primary, Last accessed on 17 February 2025) urged voters to abstain from voting, attempting to manipulate electoral participation. This example underscores the growing risk of AI-driven political disinformation. These cases are among the latest attacks leveraging generative AI technology, emphasizing the need for effective and robust detection mechanisms.

One profound consequence of the proliferation of Deepfakes is the emergence of a cognitive phenomenon termed “Impostor Bias” [3]. This bias reflects the tendency of individuals to question the authenticity of multimedia elements, driven by the awareness of AI’s ability to generate realistic forgeries. This skepticism, while protective in some cases, also undermines trust in legitimate media and digital interactions.

To counter these challenges, the field of deepfake detection has gained significant traction. Researchers have developed methods [4] to identify inconsistencies and artifacts imperceptible to the human eye but detectable using machine learning algorithms. These approaches, leveraging Convolutional Neural Networks (CNNs) [5,6], focus on analyzing spatial and temporal anomalies, ranging from pixel-level distortions to inconsistencies in biological or behavioral cues. As deepfake generation techniques evolve, so do detection strategies, creating a dynamic and continuous arms race.

The scope of forensic analysis in the deepfake domain extends beyond detection, encompassing several critical research areas. These include Deepfake Attribution and Recognition, Passive and Active Authentication, and methods designed to operate effectively in realistic, real-world scenarios.

- **Deepfake Attribution and Recognition** aims to trace the origins of synthetic content by identifying the specific models used in its creation. This involves analyzing the *traces* left by generative architectures, enabling the attribution of content to its source.
- **Passive Authentication Methods** focus on evaluating the authenticity of media through inherent characteristics, such as statistical irregularities, without requiring additional embedded data. These methods are particularly effective for retrospective analysis.
- **Active Authentication techniques**, in contrast, embed verifiable information into media during its creation, such as digital watermarks or cryptographic signatures, allowing for immediate and robust verification.
- **Realistic Scenario Detection** addresses the challenges posed by deepfake content in uncontrolled environments, such as low-resolution, compressed, or adversarially manipulated media. This is critical for practical applications where detection systems must operate under diverse and unpredictable conditions.

In this context, the authors of this paper are involved in the FF4ALL initiative (FF4ALL-Detection of Deep Fake Media and Life-Long Media Authentication) (research project line in the context of Spoke 2 “Misinformation and Fakes” funded by the Italian national PNRR and European NextGenerationEU funds), which aims to develop theoretical and practical tools for detecting and combating media counterfeits or deepfakes, tracing their origin and limiting their dissemination. This will be achieved through passive analysis techniques that operate when the content is used or disseminated and active protection methods to be adopted at the time of content creation to facilitate subsequent authentication. Table 1 summarizes the main topics of interest and research techniques on deepfakes. The project is financed within the National Recovery and Resilience Plan, Mission 4 “Education and Research”—financed by the European Union—NextGenerationEU. Further information on the project is available on the website: https://sites.unica.it/ff4all/, accessed on 15 February 2025. The project is structured into multiple Work Packages (WPs), each focusing on specific tasks related to deepfake detection and authentication:

- WP1—Deepfake Attribution and Recognition
  - Task 1.1—Deepfake Fingerprint;
  - Task 1.2—Deepfake Attribution;
- WP2—Passive Deepfake Authentication Methods
  - Task 2.1—Deepfake and Biometric Recognition
  - Task 2.2—Audio–Video Deepfake;
  - Task 2.3—Advanced Methods for Deepfake Detection;
- WP3—Deepfake Detection Methods in Realistic Scenarios
  - Task 3.1—Deepfake Detection of image-videos in the Wild
  - Task 3.2—Deepfake and Social Media;
  - Task 3.3—Detection of Deepfake Images and Videos in Adversarial Settings;
- WP4—Active Authentication
  - Task 4.1—Active Fingerprinting for Deepfake Detection and Attribution
  - Task 4.2—Authentication of Devices for the Acquisition and Processing of Content;
  - Task 4.3—Trusted Remote Media Processing on Cloud and Edge Computing Systems

Building upon our previous work [7], this extended version provides a structured comparison of deepfake detection approaches, analyzing their strengths and weaknesses. Additionally, it introduces a more detailed discussion of the deepfake generation process, the methodology behind dataset creation, and an improved presentation of the FF4ALL project’s objectives. Furthermore, this version expands the discussion on open challenges in deepfake forensics, identifying key research gaps and future directions for improving detection robustness, and forensic applicability. In addition, unlike previous studies that focus on isolated aspects of deepfake detection, this work provides an integrated perspective on forensic challenges, real-world constraints, and adaptive methodologies.

In particular, Section 2 provides a brief introduction to the deepfake generation process. Section 3 categorizes and examines current deepfake detection algorithms, providing a detailed analysis of their advantages and disadvantages, while Section 4 introduces the most used deepfake datasets in state of the art. However, as deepfake techniques continue to advance, many of the existing methods struggle to adapt to new forms of manipulation, such as domain shifts or previously unknown attack patterns. To address this limitation, Section 5 investigates continuous learning techniques, which enable detection models to evolve alongside deepfake technologies and adapt to emerging threats.

Although continual learning enhances the adaptability of detection models, it also introduces increased complexity, raising critical questions about the interpretability of these systems. In forensic investigations, explainability is crucial for ensuring the reliability, trustworthiness, and accountability of AI-driven detection tools. Building on this, Section 6 explores methods to improve the interpretability of detection models, ensuring that these tools can be understood and validated by forensic experts.

Beyond the detection of manipulations, however, tracing the origins of synthetic media is equally critical for holding perpetrators accountable and understanding the generative processes behind deepfakes. Section 7 extends the discussion by delving into deepfake attribution and model fingerprinting techniques, which aim to identify the sources of synthetic media and the generative models responsible for their creation.

To provide a broader perspective, Section 8 examines passive deepfake authentication methods, which emphasize non-intrusive approaches to verifying media authenticity. Section 9 focuses on the practical challenges of detecting manipulated content in realistic scenarios, such as highly compressed media commonly found on social networks. Section 10 complements these discussions by addressing active authentication techniques, such as cryptographic signatures, which proactively embed trust and ensure media authenticity at the point of creation.

A general discussion is provided in Section 11, synthesizing the insights from all methodologies and exploring their interconnections. Finally, Section 12 evaluates the strengths and limitations of the methodologies discussed, outlines future directions, and provides a comprehensive perspective on advancements in deepfake media forensics.

## 2. Deepfake Generation Process

Generative models are a class of machine learning algorithms designed to learn the underlying distribution of a given dataset and generate new samples that resemble the real data. Unlike discriminative models, which focus on distinguishing between different classes or making predictions, generative models aim to capture the data distribution $p_{data}$ and synthesize new samples that share similar characteristics. These models have gained significant attention in various domains, including image synthesis, text generation, music composition, and data augmentation. Two of the most prominent approaches in generative modeling are Generative Adversarial Networks (GANs) and Diffusion Models (DMs). While both methodologies aim at producing high-quality synthetic data, they employ fundamentally different principles and learning paradigms. GANs rely on an adversarial game between two neural networks, while diffusion models leverage a sequential denoising process to learn the data distribution. Some aspects of this framework will be examined in the next subsection.

**Figure 1:**
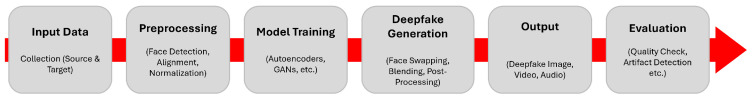
Generic diagram of the deepfake creation process.

In general, deepfake creation involves a multi-step pipeline that leverages advanced machine learning techniques to synthesize highly realistic media. Figure 1 shows a generic deepfake creation process, which begins with input data collection, in which source and target videos or images are collected. This is followed by preprocessing, which includes face detection, alignment, and normalization to ensure that the data is suitable for training. The model training phase employs neural network architectures such as autoencoders and GANs (Generative Adversarial Networks) to learn and replicate facial features and expressions. Once the model is trained, the deepfake generation step involves face swapping, blending, and post-processing to produce a seamless and realistic deepfake. The final output is a deepfake image, video, or audio. The process concludes with evaluation, where the quality of the deepfake is assessed, and any artifacts or inconsistencies are identified. This structured workflow highlights the key stages and techniques involved in deepfake creation, providing a comprehensive understanding of the process.

### 2.1. Generative Adversarial Networks (GANs)

**Figure 2:**
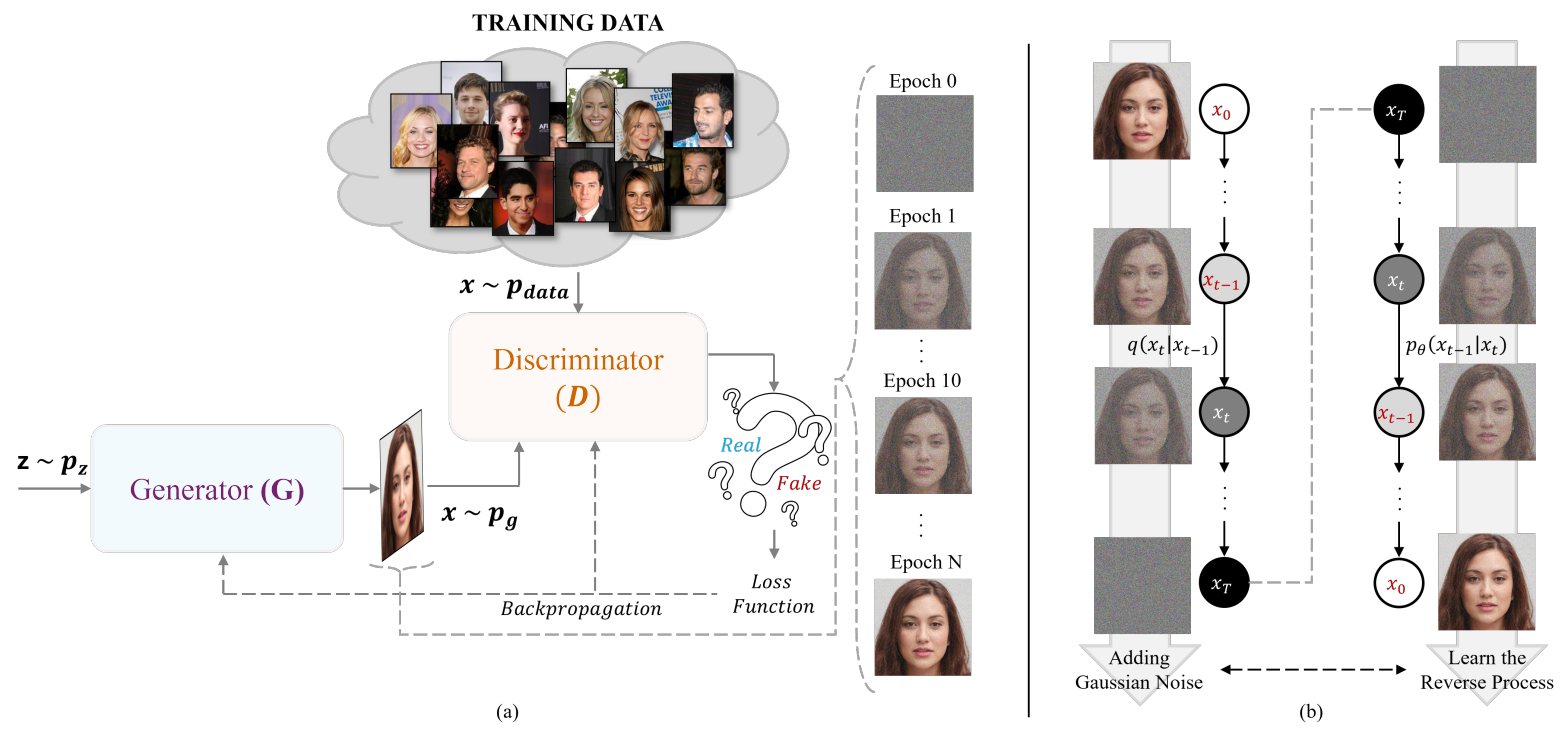
(**a**) The GAN framework consists of a Generator (*G*) that creates synthetic data $\bar{x}$ from random noise *z*, aiming to learn the data distribution $p_{data}$, and a Discriminator (*D*) that distinguishes between real and generated data. Both models are trained simultaneously in an adversarial manner; (**b**) The Diffusion Model [8] uses a fixed Markov chain to add Gaussian noise to data, approximating the posterior distribution $q(x_{t}|x_{t-1})\;\mathrm{for}\;t=1,\ldots ,T$. The goal is to learn the reverse process $p_{\theta }\left(x_{t-1}|x_{t}\right)$ to generate data by reversing the noise-adding chain, where $x_{1},\ldots ,x_{T}$ are latent variables with the same dimensionality as $x_{0}$.

The GAN framework [1] consists of two neural networks trained in a competitive manner (Figure 2a):

The Generator (G), which takes a random noise vector *z* sampled from a prior distribution (e.g., Gaussian or uniform) and maps it to a data sample $\bar{x}$. The objective of *G* is to learn the underlying distribution of the training data and produce samples that are indistinguishable from real ones. The Discriminator (D), which acts as a binary classifier, distinguishing between real data from the training set and synthetic data generated by *G*. Mathematically, the objective function of a GAN can be expressed as a minimax game (Equation (1)):

(1) $$\min_{G}\max_{D}V\left(D,G\right)=E_{x∼p_{data}\left(x\right)}\left[\log D\left(x\right)\right]+E_{z∼p_{z}\left(z\right)}\left[\log\left(1-D\left(G\left(z\right)\right)\right)\right]$$

where $p_{data}\left(x\right)$ represents the real data distribution, and $p_{z}\left(z\right)$ is the prior distribution used to sample the input noise for *G*. The training process involves an iterative optimization where:

- *D* is optimized to maximize its ability to differentiate between real and fake samples.
- *G* is optimized to minimize the ability of *D* to correctly classify generated samples as fake.

Through this adversarial process, the generator progressively improves, producing increasingly realistic samples. GANs have been widely applied in tasks such as image generation (e.g., StyleGAN, BigGAN), super-resolution, and domain adaptation. However, they often suffer from challenges like mode collapse, where *G* generates only a limited variety of samples, and training instability due to the adversarial nature of optimization.

### 2.2. Diffusion Models

Diffusion Models (DMs) [8] represent a fundamentally different generative paradigm, inspired by nonequilibrium thermodynamics and probabilistic modeling (Figure 2b). They operate by progressively corrupting the input data through a forward process and then learning to reverse this degradation to reconstruct samples from noise.

The forward process is defined as a fixed Markov chain that gradually adds Gaussian noise to the data (Equation (2)):

(2) $$q(x_{t}|x_{t-1})\;\mathrm{for}\;t=1,\ldots ,T$$

where $x_{1},\ldots ,x_{T}$ represent latent variables with the same dimensionality as the original data $x_{0}$. This process eventually transforms the input data into a nearly isotropic Gaussian distribution.

The training objective of diffusion models is to learn the reverse process (Equation (3)):

(3) $$p_{\theta }\left(x_{t-1}|x_{t}\right)\;\mathrm{for}\;t=1,\ldots ,T$$

which enables the model to generate new samples by starting from pure noise and gradually refining the data through a learned denoising process.

Unlike GANs, which rely on adversarial training, diffusion models optimize a likelihood-based objective, often implemented through denoising score matching or variational inference. This makes them more stable to train and less prone to mode collapse. Recent advances in diffusion models, such as Denoising Diffusion Probabilistic Models (DDPMs) and Score-Based Generative Models, have demonstrated state-of-the-art performance in image synthesis, rivaling and sometimes surpassing GANs in quality and diversity.

## 3. Deepfake Detection

**Figure 3:**
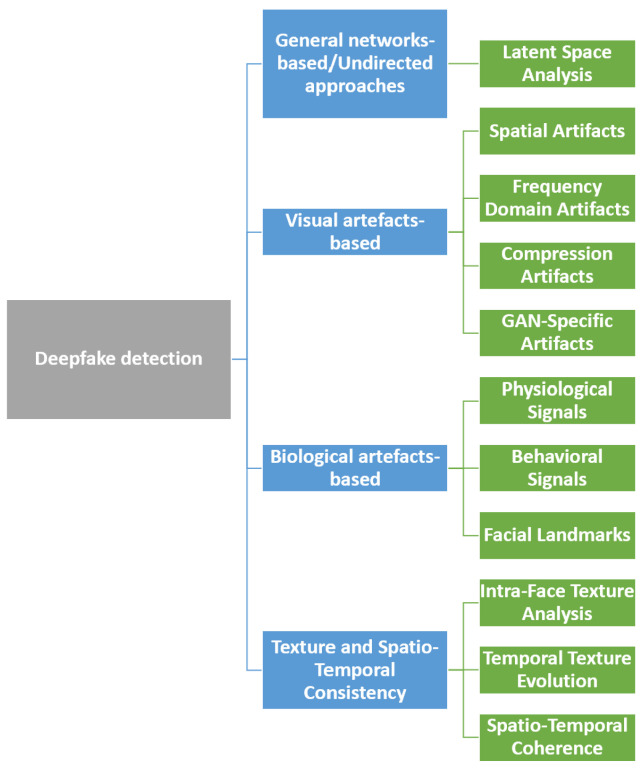
Taxonomy of Deepfake Detection Methods. Detection techniques are categorized into four primary groups: general network-based approaches, methods focusing on visual artifacts, approaches targeting biological inconsistencies, and techniques leveraging texture and spatio-temporal consistency. Each category addresses specific characteristics of manipulated content.

Ensuring the authenticity of digital content is a critical challenge in multimedia forensics as deepfake technology continues to evolve and produce increasingly realistic synthetic media. Detecting manipulated content is essential to mitigate the risks of misinformation, identity fraud, and media integrity threats [9] while also serving as the foundation for forensic analysis, attribution, and authentication. This section provides a structured overview of detection methodologies, ranging from handcrafted forensic techniques to modern data-driven approaches. Early forensic approaches focus on detecting explicit spatial inconsistencies, such as unnatural lighting, pixel-level anomalies, and compression artifacts [10]. While these methods remain valuable, they often struggle against highly sophisticated manipulations. To overcome these limitations, deep learning models exploit data-driven patterns, learning to recognize subtle inconsistencies imperceptible to the human eye [11]. A comprehensive exploration of these methodologies is presented below, highlighting their principles, technical approaches, limits, and contributions to advancing the field of deepfake detection (Figure 3).

### 3.1. Undirected Approaches

Undirected approaches harness the adaptability of deep learning models to autonomously learn features from data rather than relying on predefined artifacts. This capability is critical for addressing novel manipulation techniques, which continue to emerge in the evolving landscape of deepfake technology. Deep learning-based techniques often rely on architectures such as Convolutional Neural Networks (CNNs) and autoencoders, which are well-suited for extracting features from spatial and temporal domains [12]. Adaptability in detecting manipulations stems from the ability of these methods to learn hierarchical representations directly from data. Central to this process are latent representations, compact and structured spaces generated during training that capture high-level patterns often imperceptible in raw data. Leveraging these latent spaces enables deep learning models to achieve greater flexibility compared to manually designed features, improving generalization to novel and unseen manipulations. However, their effectiveness heavily depends on their ability to capture the variability of forgery types comprehensively. When the latent space is insufficiently representative, models struggle to generalize beyond the training data. Yan et al. [13] addressed this limitation through Latent Space Data Augmentation (LSDA), a technique designed to enrich the latent space by interpolating diverse samples, thereby enhancing the generalization capabilities of deep learning-based detectors. Another limitation of latent space-based methods is their high computational demands and reliance on extensive labeled datasets, which are resource-intensive and time-consuming to develop [14]. Transfer learning offers a potential solution by enabling models to adapt pre-trained latent representations to new tasks with a reduced need for extensive and fully annotated datasets [15].

### 3.2. Visual Artifact-Based Detection

**Figure 4:**
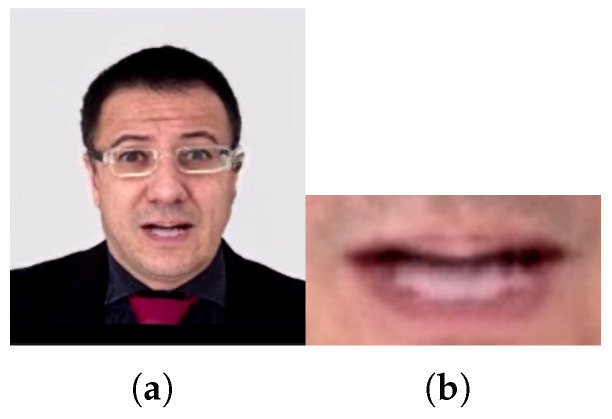
Example of deepfake detection features. The full face (**a**) exhibits visual artifacts, such as unnatural pixel formations around the facial features (e.g., glasses and skin edges). These are indicative of irregularities introduced by generative algorithms or compression distortions. The zoomed-in region (**b**) around the mouth shows potential texture inconsistencies, such as unnatural blending of lip textures and a lack of smooth transitions typical of natural skin and lip patterns.

The process of creating deepfake content frequently introduces subtle anomalies within the visual data, often referred to as artifacts, such as irregular pixel arrangements, distortions along edges, or inconsistencies in the spectral domain (Figure 4). Methods that focus on visual artifact detection leverage these anomalies as indicators of synthetic manipulation.

Spatial artifacts are one of the earliest and most intuitive indicators of manipulated media. They include unnatural pixel formations, edge inconsistencies, lighting mismatches, and shadow anomalies that arise due to imperfections in generative algorithms [16]. For instance, deepfake algorithms often struggle to maintain consistent lighting across a manipulated face, resulting in subtle but detectable anomalies around critical regions such as the eyes, mouth, and face contours. Frequency domain analysis provides another robust framework for detecting deepfakes by identifying anomalies in the spectral characteristics of images and videos, which are often imperceptible in the spatial domain [17]. Methods based on Fourier transforms and wavelet decomposition have proven effective in uncovering these inconsistencies, leveraging the distinct frequency patterns observed in synthetic media [18,19]. The ability to isolate and analyze frequency-specific features enhances the detection of manipulations, even under challenging conditions such as compression or resolution loss [20].

The ability to isolate and analyze frequency-specific features enhances the detection of manipulations, even under challenging conditions such as compression or resolution loss [20], which are common in deepfake content shared on social media. In fact, lossy compression algorithms, such as JPEG and MPEG, obscure subtle visual cues essential for detection, complicating the identification of deepfakes. At the same time, these compression processes introduce distinctive patterns that specialized models can exploit to reveal discrepancies [21]. For instance, Gao et al. (2024) proposed a High-Frequency Enhancement (HiFE) network that leverages the Discrete Cosine Transform (DCT) and the Discrete Wavelet Transform (DWT) to recover high-frequency details adaptively lost during compression, significantly improving detection performance on highly compressed deepfake content [22].

Lastly, GAN-specific artifacts focus on detecting traces unique to generative adversarial networks. For example, GANs often produce checkerboard patterns or exhibit anomalies in low-frequency regions, which can serve as reliable indicators of manipulation [23]. Although visual artifact-based methods are computationally efficient and effective in many scenarios, their reliance on spatial details may still pose challenges. Integrating frequency-specific analysis could offer a way to overcome these limitations, improving detection robustness against compression and resolution loss.

### 3.3. Biological Artifact-Based Detection

Biological artifact-based detection methods exploit inconsistencies in human physiology and behavior, aspects that deepfake algorithms struggle to replicate accurately. Facial regions are particularly suitable for this type of analysis due to subtle physiological and behavioral cues [24]. Physiological signals, such as variations in skin tone caused by blood flow or subtle muscle movements, often appear inaccurate or entirely absent in deepfake content. Techniques that analyze these signals, such as remote photoplethysmography (rPPG), can identify deviations from natural biological patterns by examining heart rate dynamics captured in facial videos [25]. Behavioral cues, including eye-blinking frequency, gaze direction, and lip synchronization, provide another layer of analysis. Deepfake algorithms frequently fail to maintain the natural temporal dynamics of these behaviors, leading to detectable anomalies. For instance, inconsistencies in the display of emotions, measured through valence and arousal, have been shown to indicate synthetic content [26]. Similarly, irregular or unnatural blinking frequency in deepfake videos represents a well-documented artifact, as generative models often fail to replicate natural blinking patterns consistently [27]. Facial landmarks also play a critical role in detecting manipulations. Methods that analyze facial features’ geometric relationships and alignments can identify discrepancies typical of face-swapping techniques. Deviations in the proportions or placements of key landmarks serve as reliable indicators of forgery [23]. Biological artifact-based approaches are highly effective because they target features deeply rooted in natural human physiology and behavior, which are difficult for even the most advanced generative models to replicate. However, these methods often require high-resolution input and can struggle when faces are partially occluded or poorly lit.

### 3.4. Texture and Spatio-Temporal Consistency-Based Detection

Texture and spatio-temporal consistency-based detection focus on fine-grained patterns and temporal coherence within media content. These approaches are particularly effective for video Deepfakes, where the dynamic nature of the content poses additional challenges for manipulation algorithms. Intra-face texture analysis examines inconsistencies across different regions of the same face [28]. For instance, face-swapping manipulations often blend skin textures from disparate sources, creating visible mismatches between inner and outer facial regions. Advanced image processing and deep learning techniques can effectively detect these discrepancies [29]. Temporal texture evolution extends this analysis by evaluating texture consistency over successive video frames [30]. Authentic videos typically display smooth and continuous texture transitions, while manipulated content frequently exhibits abrupt or unnatural changes that expose synthetic origins [31]. Spatio-temporal coherence methods assess the synchronization between various modalities, such as facial movements, gestures, and audio [32]. Discrepancies between speech and lip movements or misalignment between gestures and facial expressions are reliable forgery indicators. Combining these techniques provides a robust framework for identifying even sophisticated video Deepfakes. However, computational intensity and the need for high-quality video input can limit their practicality in real-world applications.

## 4. State-of-the-Art Deepfake Datasets

In this section, we present an overview of the most widely used datasets in state-of-the-art research on deepfake detection. These datasets span various modalities, including image, audio, video, and multimodal data, reflecting the multifaceted nature of the deepfake detection problem. The datasets vary in composition, with some containing exclusively synthetic data, others comprising only real data, and some offering a combination of both. The choice of dataset depends on the specific objectives of the analysis, as each corpus can be utilized for training, testing, or evaluating the generalization capabilities of deepfake detection models. Cross-dataset evaluation is particularly critical, as it enables the assessment of model robustness and generalization across diverse data sources, a key requirement in multimedia forensics. This ensures that detectors perform reliably not only on data similar to the training set but also on entirely different datasets.

### 4.1. Image Datasets

**WildFake [33]** is a large-scale dataset designed to facilitate the detection of AI-generated images. It comprises a diverse collection of fake images sourced from open communities and multiple generative models, ensuring a rich variety of content and styles. The dataset is hierarchically organized, categorizing images based on generative model types, architectures, weights, versions, and release timelines. With over 3.6 million images (2.6 M fake and 1 M real), WildFake provides a comprehensive resource for training and evaluation. Real images are curated from various open datasets to ensure realistic comparisons.

**Artifact [34]** is a dataset designed to evaluate the generalizability and robustness of synthetic image detectors. It includes a diverse range of images across multiple object categories, such as human faces, animals, vehicles, places, and artworks. The dataset contains over 2.4 million images, with 1.5 million synthetic images generated using 25 different methods, including GANs, diffusion models, and other generative techniques. To simulate real-world conditions, the dataset incorporates social media-related impairments like compression, resizing, and cropping. Artifact introduces a novel multi-class classification approach, distinguishing between real images, fake images from known generators, and fake images from unseen generators. Additionally, it employs a Filter Stride Reduction (FSR) technique to preserve generator artifacts despite image degradation.

**DIFF [35]** is a large-scale dataset specifically designed for detecting diffusion-generated facial forgeries. It contains over 500,000 images synthesized using 13 state-of-the-art diffusion models under four conditions: Text-to-Image (T2I), Image-to-Image (I2I), Face Swapping (FS), and Face Editing (FE). The dataset is curated using 30,000 textual and visual prompts to ensure high-fidelity and semantically consistent forgeries. Real images from 1070 celebrity identities are included to ensure diversity across gender and age groups. Experiments reveal that both human and automated detectors struggle with these forgeries, underscoring the challenges in detection.

**StyleGAN-XL [36]** is an extension of the StyleGAN family, designed to generate high-quality images on diverse and unstructured datasets, particularly ImageNet. Unlike its predecessors, StyleGAN [37] and StyleGAN2 [38], which excelled at synthesizing human faces and structured datasets, StyleGAN-XL incorporates Projected GAN training and a progressive growing strategy to enhance stability and scalability. The model achieves state-of-the-art performance in large-scale image synthesis, generating high-resolution images up to 1024 × 1024. It also introduces classifier guidance for improved class-conditional generation. StyleGAN-XL overcomes the scalability issues of previous models, offering better sample diversity, generalization, and image fidelity. The dataset provides new benchmarks for GAN-based image generation and detection, positioning it as a strong alternative to diffusion models.

### 4.2. Audio Datasets

**ASVspoof 2019 [39,40]** is a benchmark dataset for synthetic speech detection, containing both real and synthetic audio tracks from 78 speakers (33 male, 45 female) based on the VCTK corpus. The dataset was released for a challenge focused on Automatic Speaker Verification (ASV). Its Logical Access (LA) partition contains deepfake data, which is divided into training, development, and evaluation subsets. The training and development subsets include synthetic speech generated using six algorithms, while the evaluation subset includes 13 techniques, only two of which overlap with the training set. This structure allows for open-set evaluation, assessing detectors on unseen synthesis algorithms. The dataset is highly imbalanced, with synthetic data significantly outnumbering real data, a factor that must be considered during model training.

**ASVspoof 2021 [41]** is an updated version of ASVspoof 2019, released for a subsequent challenge. It includes three evaluation partitions (LA, PA, and DF), with no new training or development data. The deepfake (DF) partition introduces distortions through lossy codecs commonly used in media storage, simulating real-world conditions.

**TIMIT-TTS [42]** is a synthetic speech dataset generated using 12 different TTS methods, based on the VidTIMIT corpus. It includes four partitions corresponding to different post-processing pipelines: clean, augmented, Dynamic Time Warping (DTW), and DTW+augmented. Each partition contains 19,780 tracks, totaling nearly 80,000 synthetic speech signals. This dataset can be used independently or combined with VidTIMIT and DeepfakeTIMIT for multimodal research.

**AISEC “In-the-Wild” [43]** is a synthetic speech dataset designed to replicate real-world conditions. It includes 38 h of audio clips (17 h fake, 21 h real) featuring English-speaking celebrities and politicians. The fake clips are derived from publicly available deepfake content, while real clips are manually collected from sources like podcasts and speeches.

**FakeOrReal [44]** is a speech dataset containing over 198,000 utterances, including both real and synthetic tracks. The synthetic data are generated using TTS methods, while real data are sourced from open datasets and platforms like TED Talks and YouTube.

**Purdue Speech Dataset [45]** includes 25,000 synthetic speech tracks generated using five advanced diffusion model-based voice cloning methods (ProDiff, DiffGAN-TTS, ElevenLabs, UnitSpeech, and XTTS). Real speech data are sourced from LJSpeech [46] and LibriSpeech [47].

### 4.3. Video and Multimodal Datasets

**DeepfakeTIMIT [48]** is a video deepfake dataset derived from the VidTIMIT corpus [49], which consists of real audio–video recordings of 43 individuals, each reciting short sentences, resulting in a total of 430 videos. The DeepfakeTIMIT dataset focuses exclusively on visual forgeries, leaving the audio component unaltered. The fake video frames are generated using a GAN-based approach adapted from Faceswap [50]. The dataset includes deepfakes of 32 subjects, available in two versions: Low Quality (LQ) and High Quality (HQ), differing in frame resolution. In total, the dataset comprises 640 videos, with 320 videos in each quality category.

**FaceForensics++ [51]** is a widely used visual-only deepfake dataset containing 5000 videos generated using four distinct deepfake generation methods. The dataset is based on 1000 real YouTube videos and includes two partitions with different levels of compression, quantified by the Quantization Parameter (QP). Specifically, the partitions correspond to QP values of 23 and 40, where a higher QP indicates lower video quality.

**DFDC [52]** (DeepFake Detection Challenge) is a multimodal deepfake dataset comprising nearly 120,000 videos, of which 100,000 are labeled as fake and the remaining as real. The videos are organized into 50 folders, each containing a set of real videos and their corresponding fake derivatives. Each folder features videos of different subjects. While the dataset does not provide separate labels for the authenticity of individual modalities (audio or video), a video is labeled as fake if either the audio or video component is manipulated. Although the majority of the videos are visual-only forgeries, a subset of videos in folders 45 to 49 includes falsified audio in addition to potential visual manipulations.

**FakeAVCeleb [53]** is a multimodal deepfake dataset constructed from 500 real videos extracted from the VoxCeleb2 corpus [54]. These real videos serve as the basis for generating approximately 20,000 deepfake videos using various deepfake generation techniques. The subjects in the videos are English speakers with diverse ethnicities and accents. The deepfake video frames are created using Faceswap [50] and FSGAN [55], while the synthetic audio is generated using Real-Time Voice Cloning (RTVC) [56]. To ensure synchronization between the video frames and audio, Wav2Lip [57] is applied. The dataset is divided into four distinct partitions: *Real Video–Real Audio*, *Fake Video–Real Audio*, *Real Video–Fake Audio*, and *Fake Video–Fake Audio*, enabling comprehensive studies on multimodal deepfake detection.

**DeepSpeak 1.0 [58]** is a large-scale dataset featuring real and deepfake footage of individuals speaking and gesturing in front of webcams. The dataset includes 17 h of real video footage from 220 diverse individuals and over 26 h of deepfake footage generated using state-of-the-art face-swap and lip-sync techniques. The deepfake videos incorporate both natural and AI-generated voices, providing a realistic representation of advanced deepfake technologies. The authors anticipate releasing future versions of the dataset, incorporating updated deepfake methods to reflect evolving technological advancements.

## 5. Continual Learning for Deepfake Detection

Deep learning models face significant challenges in handling dynamically evolving data distributions, particularly in areas such as audio deepfake detection. Traditional deep learning approaches assume that training and testing data share a static distribution, which is unrealistic in scenarios where new spoofing attacks emerge regularly [59]. Consequently, these models fail to generalize to unseen data and are highly susceptible to catastrophic forgetting when fine-tuned on new tasks [60]. Catastrophic forgetting occurs because gradient-based optimization overwrites parameters optimized for earlier tasks, thereby degrading the model’s performance on those tasks.

Continual learning (CL) provides a framework to mitigate this issue by enabling models to learn new tasks sequentially without forgetting previously learned ones. The stability–plasticity dilemma is central to CL, as it balances the model’s ability to retain past knowledge (stability) while acquiring new information (plasticity) [61,62]. This balance ensures that the model can adapt to evolving tasks while maintaining performance on earlier ones, making CL essential for dynamic learning scenarios. To achieve this balance, CL leverages strategies such as regularization-based approaches, experience replay, and architectural modifications, ensuring models remain adaptable while mitigating forgetting.

### 5.1. Replay-Based Methods

Replay-based methods are among the most effective strategies for continual learning, particularly in scenarios with evolving tasks and data distributions. These methods mitigate catastrophic forgetting through the use of a memory buffer that stores past samples, which are revisited periodically during training on new tasks [63]. Incorporating historical data reinforces prior knowledge while allowing the model to adapt to new information. Replay methods vary in their implementation, with some utilizing exact sample storage (e.g., Experience Replay) [64] and others employing generative models to recreate past samples (e.g., Generative Replay) [65]. Selective sampling techniques are often integrated to optimize memory usage, ensuring that samples contributing the most to knowledge retention are prioritized. In audio deepfake detection, replay-based methods enhance the model’s ability to detect both older and emerging deepfake attacks. Reintroducing samples of earlier spoofing techniques during training allows the model to generalize across diverse spoofing patterns and maintain high detection accuracy across evolving scenarios. This strategy ensures a balanced representation of past tasks, supporting robust generalization and effective adaptation in dynamic environments.

Ma et al. [66] introduced the replay based method known as Detecting Fake Without Forgetting (DFWF) method, a robust framework designed to mitigate catastrophic forgetting and enable efficient audio deepfake detection. DFWF integrates Learning without Forgetting (LwF) and Positive Sample Alignment (PSA) to preserve past knowledge while incrementally adapting to new spoofing types. LwF employs a knowledge distillation loss to align the output probabilities of the current and original models, ensuring the retention of prior knowledge and the model’s ability to recognize previously encountered spoofing attacks. PSA focuses on maintaining consistent representations of genuine audio by aligning embeddings across tasks using a cosine similarity loss, enabling the model to effectively differentiate genuine audio from new spoofing attacks. These components are combined into a composite loss function that balances knowledge retention and adaptation. DFWF achieved an Average Equal Error Rate (AvgEER) reduction of 81.63% compared to fine-tuning, maintaining robust performance on both new and old tasks of ASVspoof 2019. Nguyen et al. [67] proposed a Transformer-based model with a continual learning method for incremental training, focusing on unseen spoofing attacks. The approach employs few-shot learning and embedding similarity loss to adapt to new tasks with minimal labeled data. Using the ASVspoof datasets and over 2 million synthetic samples, the model achieved 90–95% AUC on unseen datasets, demonstrating strong generalization capabilities. By combining few-shot learning with replay strategies, this method offers a robust and scalable approach for tackling evolving audio deepfake threats.

### 5.2. Regularization-Based Methods

Regularization-based continual learning methods mitigate catastrophic forgetting by adding constraints to the loss function, ensuring that updates to the model parameters do not interfere significantly with knowledge learned from previous tasks [68]. These techniques identify and preserve critical parameters through mechanisms such as Elastic Weight Consolidation (EWC) or knowledge distillation, which penalize large deviations in key weights or align output distributions between tasks. This approach allows the model to retain prior knowledge while adapting to new data, balancing stability and plasticity. Dong et al. [69] introduced the Continual Audio Defense Enhancer (CADE) built by integrating regularization techniques with replay strategies to enhance robustness in audio deepfake classification. CADE employs Knowledge Distillation Loss, which ensures that the current model’s predictions remain aligned with its previous outputs, and Positive Sample Alignment (PSA) loss, which maintains consistent embeddings of genuine audio across tasks. Unlike purely replay-based methods, CADE leverages regularization to further mitigate forgetting and improve performance. Evaluations conducted on the ASVspoof2019 dataset demonstrate that CADE achieves lower EERs compared to baseline methods, effectively retaining knowledge of previously seen spoofing attacks while adapting to new ones. This hybrid approach highlights the strength of combining replay and regularization for balanced and scalable continual learning in dynamic environments.

### 5.3. Architectural-Based Methods

Architectural strategies dynamically modify the structure of neural networks to balance the retention of old knowledge and the acquisition of new information. Methods such as Orthogonal Weight Modification (OWM) prevent interference with previously learned tasks by projecting gradient updates onto an orthogonal subspace, ensuring minimal disruption to past knowledge [70]. This type of technique enhance adaptability and robustness, particularly for sequential learning tasks like audio deepfake detection, by maintaining performance on previously learned tasks.

The authors of [71] developed the Radian Weight Modification (RWM) method, a novel architectural approach for continual learning that categorizes classes based on feature distribution similarity. Compact classes (e.g., genuine audio) are treated with minimal gradient updates to retain stability, while dispersed classes (e.g., fake audio) receive orthogonal updates to prevent interference with prior knowledge. The method also incorporates a self-attention mechanism to dynamically adjust gradient directions, ensuring efficient retention of previous tasks and adaptation to new data. Unlike the RWM method, which focuses on radial modifications of gradient directions, the authors of [72] introduced Dynamic Class Rebalancing (DCR) as part of a framework designed to handle imbalanced class distributions dynamically. The approach integrates SincNet as a teacher model and LightCNN as a student model, combining Feature Distillation (FD) with DCR. FD transfers discriminative features from SincNet to LightCNN, ensuring robust feature representation in the student model, while DCR categorizes classes based on feature similarity and adjusts learning strategies dynamically. This allows for enhanced adaptability to evolving data while retaining prior knowledge. The authors of [73] introduced the EVDA benchmark, designed to evaluate various continual learning methods for audio deepfake detection across diverse datasets and conditions. The benchmark includes datasets such as ASVspoof2015, ASVspoof2019, and FoR, alongside new challenges like real-world scenarios and cross-lingual datasets. Key continual learning methods evaluated include OWM, RWM, and Regularized Adaptive Weight Modification (RAWM). Among these, OWM dynamically adjusts weight updates to minimize interference with previously learned tasks, while RWM optimizes gradients for stability across evolving datasets. The results show that replay-based and architectural methods consistently outperform baseline approaches in maintaining low EER and adapting to new tasks, demonstrating the benchmark’s utility for advancing robust and adaptive audio deepfake detection.

Continual learning approaches provide a robust framework for addressing the dynamic and evolving nature of audio deepfake detection. By leveraging strategies such as replay, regularization, and architectural modifications, these methods effectively mitigate catastrophic forgetting while enabling models to adapt to unseen spoofing attacks. Replay-based methods, like DFWF and Transformer-based models, enhance generalization by revisiting past knowledge and incorporating incremental learning for emerging threats. Regularization techniques, exemplified by CADE, combine knowledge distillation and embedding alignment to preserve stability across tasks while retaining flexibility for new data. Architectural strategies, such as RWM and DCR, dynamically adjust learning pathways, offering adaptability to imbalanced and complex distributions.

Future research in continual learning for audio deepfake detection could explore hybrid frameworks that integrate multiple strategies, such as combining replay with advanced architectural modifications to enhance scalability or applying continual learning techniques only to limited portions of the models [74]. The use of generative models for creating realistic past samples and novel benchmarks, like EVDA, can push the boundaries of evaluating and improving performance in cross-lingual scenarios. Additionally, integrating self-supervised learning for feature extraction and multi-modal data, including audio and visual signals, could further enhance robustness against sophisticated deepfake techniques.

## 6. Explainability and Interpretability in AI Forensics

While continual learning improves the adaptability of detection models, it also adds complexity, posing significant challenges regarding their interpretability. In forensic investigations, ensuring explainability is essential to maintaining the reliability, credibility, and accountability of deepfake detection systems. In fact, deepfake detection systems must provide clear and interpretable decision processes to be admissible in legal and high-stakes scenarios. Explainable and interpretable AI is therefore not merely a desirable feature, it is essential. The following points illustrate why explainability and interpretability are critical in forensic contexts:

- *Enhanced Trust and Credibility:* Forensic evidence underpins legal and judicial decisions, making transparency a non-negotiable requirement. When deepfake detection systems clearly reveal which features, audio segments or images parts contributed to their predictions, forensic experts, legal practitioners, and jurors can better understand and trust the outcomes [75]. This transparency builds confidence in the system and supports the use of its outputs as credible evidence in court.
- *Accountability and Legal Defensibility:* In the courtroom, every piece of digital evidence must be defensible under rigorous scrutiny. Black-box models, despite their high predictive performance, often lack the necessary transparency [76]. Explainable AI provides a detailed trace of the decision-making process, allowing forensic analysts to demonstrate exactly how a conclusion was reached [77]. This level of traceability is critical for ensuring that evidence can be defended during cross-examinations and for establishing clear lines of accountability.
- *Identification of Biases and Error Sources:* Forensic applications demand the highest standards of accuracy and fairness. By visualizing the specific features or segments that influence model predictions, explainable AI techniques enable analysts to identify potential biases or sources of error [78]. This scrutiny is essential not only for improving the reliability of detection systems but also for ensuring that the evidence is free from hidden biases that could undermine a case.
- *Facilitating Expert Collaboration and Continuous Improvement:* Effective forensic analysis is inherently multidisciplinary, involving experts from fields such as audio engineering, computer vision experts, computer science, and law [79]. Interpretable models provide a common language for these experts by clearly explaining the inner workings of the detection system [80]. This shared understanding is vital for ongoing refinement and adaptation of the technology, particularly as new deepfake generation techniques emerge.

### 6.1. Interpretability and Explainability in Audio Deepfake Detection

Audio deepfake detection has gained significant attention in recent years, driven by the rapid advancements in synthetic audio generation technologies. While detection methods have evolved in accuracy and robustness, their lack of interpretability and explainability limits their broader adoption in high-stakes applications such as forensic analysis, media authentication, and legal proceedings [63]. Explainable Artificial Intelligence (XAI) addresses this gap by making AI models more transparent, enabling users to understand how decisions are made, which features are critical, and why certain predictions are trusted. In the context of audio deepfake detection, interpretability and explainability are crucial not only for technical refinement but also for ensuring trust, accountability, and informed decision-making in forensic applications. In forensic investigations, every decision must be both reproducible and legally defensible, making the clarity provided by explainable AI indispensable. The need for interpretability in audio deepfake detection arises from several factors:

1. *Trust and Adoption:* Black-box AI systems, while powerful, often fail to gain user trust due to their opaque decision-making processes. In forensic contexts, where evidence may be scrutinized in court, interpretability builds confidence by providing insights into how and why predictions are made [81]. This transparency is essential for the credibility of the evidence, ensuring that forensic experts and legal practitioners can understand and explain the basis of the AI’s conclusions.
2. *Debugging and Model Improvement:* Understanding model behavior helps researchers identify biases, weaknesses, or errors in the detection process, leading to targeted improvements. In forensic applications, such insights are crucial for continuously refining the system, thereby ensuring that it reliably distinguishes between genuine and synthetic audio even under diverse and challenging conditions.
3. *Accountability in Sensitive Applications:* In high-stakes settings such as legal proceedings and forensic analysis, explainability ensures that decisions are defensible and grounded in understandable reasoning [82]. This traceability is vital in court, where experts must justify the methods and evidence used in reaching a conclusion. Clear explanations not only support the legal validity of the findings but also facilitate cross-examination by providing a transparent decision-making pathway.
4. *Generalization and Robustness:* Explainable models are designed to focus on meaningful, interpretable features rather than spurious correlations [83], inherently enhancing their ability to generalize across diverse datasets. These models exhibit promising performance when exposed to new, unseen deepfakes generated by techniques not encountered during training. Preliminary evaluations indicate that emphasizing core audio features such as critical frequency bands and temporal patterns enables the models to maintain reliability and fairness even as the characteristics of synthetic audio vary widely. This robustness is crucial in forensic scenarios, where audio samples differ significantly in quality, origin, and attack strategy. Moreover, the interpretability framework not only clarifies the model’s decision-making process but also serves as a diagnostic tool, enabling researchers to identify and adapt to novel deepfake patterns as they emerge [84]. A systematic analysis of feature attributions across diverse datasets helps identify potential vulnerabilities and address them effectively, reinforcing the model’s applicability in dynamic, real-world settings.

### 6.2. Methods for Interpretability and Explainability

Various interpretability techniques have been adapted to deepfake detection, focusing on feature importance, visualization, and prototype-based reasoning. Below, we explore these state-of-the-art methods in detail, along with their applications.

- *Feature Attribution Methods:*
  Feature attribution techniques identify which parts of the input data contribute most to the model’s predictions. In audio deepfake detection, this often involves analyzing spectrograms or waveforms to highlight critical regions that differentiate real from synthetic audio. In image detection, this typically involves analyzing pixel importance through methods such as saliency maps, Grad-CAM, or SHAP to highlight key regions that influence the classification decision. Lim et al. [85] used layer-wise relevance propagation (LRP) and Deep Taylor Decomposition to explain predictions in spectrogram-based detection models. These techniques highlight frequency bands and temporal regions most influential in the model’s decisions, thereby providing forensic experts with insight into the acoustic features that distinguish genuine audio from deepfakes. Similarly, Yu et al. [86] employed SHAP to calculate feature attribution values in a lightweight machine learning framework. By visualizing these values on spectrograms, they identified critical high-frequency amplitude patterns and harmonics, thus improving both transparency and trust.
- *Attention Mechanisms for Explainability:*
  Attention mechanisms provide a natural form of interpretability by highlighting parts of the input data that the model focuses on during decision-making. Channing et al. [87] implemented an attention roll-out mechanism for Transformer-based classifiers, visualizing attention weights across audio segments. This approach not only reveals the model’s focus but also pinpoints critical regions in the audio data that contribute most to classification, thereby enhancing both accuracy and transparency in forensic examinations.
- *Prototype-Based Interpretability:*
  Prototype-based methods enhance interpretability by associating model decisions with specific, interpretable prototypes. Ilyas et al. [88] introduced prototype learning to align discriminative features with interpretable prototypes. Although primarily applied to visual deepfake detection, this approach can be adapted to audio. By associating specific characteristics (e.g., pitch or timbre for audio, texture, edges, or facial landmarks for images) with prototypes representing real or fake categories, forensic experts can more easily understand and communicate the model’s reasoning.
- *Lightweight and Explainable Frameworks:*
  In resource-constrained environments, lightweight frameworks with built-in explainability offer a practical solution. Bisogni et al. [89] utilized hand-crafted features such as spectral centroid and Mel-Frequency Cepstral Coefficients (MFCCs) combined with SHAP to create interpretable models that achieve robust detection performance. These frameworks balance accuracy and transparency, making them suitable for real-time applications and forensic scenarios where timely, explainable decisions are critical.

## 7. Deepfake Attribution and Recognition

### Deepfake Fingerprint and Attribution

The progression of deepfake generation technologies has necessitated sophisticated techniques to ensure accountability and mitigate malicious misuse. Researchers have focused on deepfake attribution, which involves identifying the specific model or architecture responsible for generating synthetic content, and ownership verification, aimed at protecting intellectual property. Deepfake attribution, also known as Deepfake Model Recognition, refers to the suite of methodologies designed to identify the specific generative model responsible for producing synthetic data [90,91]. This task involves not only recognizing the general architecture, such as GANs or diffusion models, but also attempting to estimate the unique weights of the model [92] that detail the instance of the architecture used to generate the deepfake. Current state-of-the-art (SOTA) methods have proven to be very effective in detecting deepfake content generated by GAN architectures [93,94,95] and Diffusion Models (DMs) [96,97], demonstrating the ability to specialize not only in recognizing the architecture but also in identifying the specific pattern within the creation process. Initial investigations have also been carried out in the audio domain, recognizing the generator used to generate speech deepfake signals [98].

**Figure 5:**
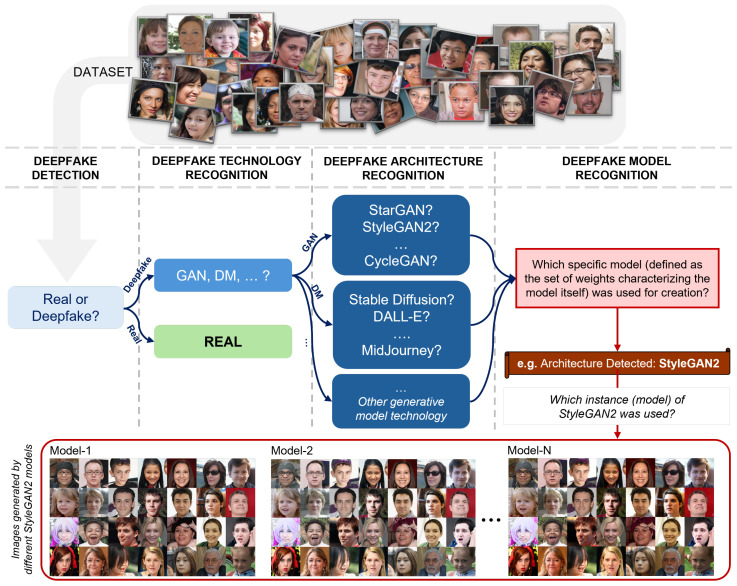
A conceptual pipeline for deepfake detection and model recognition, illustrating the process of identifying whether an image is real or synthetic, determining the generative architecture, and tracing the specific model instance used for creation.

Figure 5 provides a detailed conceptual overview of the process of detecting, classifying, and recognizing real and synthetic (deepfake) images, with a specific emphasis on identifying the generative model responsible for creating fake images. It begins with a dataset of facial images, which includes both genuine photographs and those generated by artificial intelligence. The first step involves determining whether an image is real or a deepfake. At this stage, the system focuses on differentiating between authentic content and those synthesized using generative models. For images classified as real, the process ends here. However, if an image is flagged as a deepfake, it undergoes further scrutiny to identify the specific generative technology behind it (GAN, DM, …). Next, an attempt is made to recognize the type of model used, such as GAN-based architectures (e.g., StyleGAN, StarGAN, or CycleGAN) or other generative frameworks like Stable Diffusion or DALL-E. Once the architecture is identified, the focus shifts to recognizing the precise instance of the model that produced the image. For example, if the architecture is identified as StyleGAN2, the next step is to determine the specific StyleGAN2 instance used in the image generation process. This capability is critical for forensic investigations, as it allows experts to trace the origins of synthetic content, offering insights into the tools and methodologies used.

In the context of the deepfake attribution task, several innovative SOTA approaches were proposed. Ning Yu et al. [99,100] introduced the concept of GAN fingerprints, revealing that generative adversarial networks (GANs) leave stable and unique traces in their output. These fingerprints allow for fine-grained attribution, enabling the identification of the exact GAN model used for synthesis. Their studies demonstrated the robustness of these fingerprints against adversarial perturbations, highlighting their potential in securing intellectual property and forensic investigations. Aiming to address real-world challenges, Yang et al. [101] explored architecture-level attribution in scenarios where models are fine-tuned or retrained. They proposed DNA-Det, which focuses on globally consistent fingerprints tied to the architecture, as opposed to model-specific weights. This method is particularly useful when dealing with privately trained or modified models, ensuring broader applicability in forensic settings. Sun et al. [102] introduced Contrastive Pseudo Learning (CPL) to address open-world attribution challenges. By integrating a Global–Local Voting module and soft pseudo-labeling, CPL aligns features across known and novel forgery types, enhancing model interpretability and security. A significant step forward in this field is the ability to distinguish between instances of the same architecture. For example, Guarnera et al. [90] demonstrated that the combination of a ResNET-18 model [103] with a metric learning approach [104] can achieve remarkable accuracy in distinguishing between 100 different instances of StyleGAN2-ADA [105]. This work highlights the feasibility of identifying the unique parameterization of a model instance. Furthermore, their approach showed promising results in preliminary tests involving models other than StyleGAN2-ADA, suggesting a potential for generalization across architectures.

The advent of open-world scenarios, where models encounter novel and unseen forgeries, has led to the development of benchmarks like OW-DFA++ by Sun et al. [106]. This benchmark integrates labeled and unlabeled datasets to evaluate model performance in diverse and dynamic contexts. Their proposed Multi-Perspective Sensory Learning (MPSL) framework employs multi-scale global–local feature alignment and confidence-adaptive pseudo-labeling to enhance attribution accuracy in such settings.

A robust solution for deepfake model recognition holds immense significance for intellectual property protection [107]. Such a system would enable the attribution of a specific synthetic image or video to its model owner, thereby addressing concerns over ownership and accountability in synthetic media. However, to achieve this level of precision, new strategies, and tailored metrics are required [108], particularly for cases involving high similarity between models trained with slight variations in data or hyperparameters.

In forensic applications, deepfake attribution parallels the role of camera source identification in traditional forensic investigations. Just as identifying the source camera helps trace the origin of a photograph, deepfake model recognition aims to trace synthetic media back to the specific model instance within a given architecture. This analogy underscores the critical need for advanced techniques to reliably attribute digital media to its generative source. The development of these techniques not only facilitates authenticity verification in digital forensics but also ensures accountability in cases of misuse or malicious intent. Emerging challenges in this domain include distinguishing between models trained with similar datasets, identifying models subjected to fine-tuning, and maintaining robustness against adversarial attacks [109] aimed at obfuscating model fingerprints. To address these challenges, future research can explore the integration of self-supervised learning, adversarial training, and ensemble methods that exploit multiple complementary approaches to model attribution.

## 8. Passive Deepfake Authentication Methods

In the modern era, where video calls have become a fundamental tool for global communication, ensuring the authenticity of audio and video streams is paramount. The rise of deepfake technology presents a substantial threat to the integrity of digital communication. To address this challenge, multimedia forensics researchers have developed various methods for detecting deepfakes, categorized based on the modality that they analyze.

### 8.1. Audio-Only Deepfake Detection

The rapid advancements in synthetic speech generation have increased interest in speech deepfake detection. Consequently, the scientific community has introduced a range of detection techniques employing diverse strategies and approaches [110]. Some methods focus on low-level features, looking for artifacts introduced by the generators at the signal level, while others analyze higher-level features that capture more complex aspects of speech.

An example of an artifact-based approach is presented in [111], where channel pattern noise analysis is used to secure Automatic Speaker Verification (ASV) systems against physical attacks. Similarly, the authors of [112,113,114] exploit bicoherence features based on the assumption that a genuine recording has more significant non-linearity than a fake one. Alternatively, the authors of [115,116] propose end-to-end networks trained for extracting deep features from speech, while [117] uses Mel-Frequency Cepstral Coefficient (MFCC) features as input of a Support Vector Machine (SVM) classifier. To enhance the practicality of existing detection methods in real-world scenarios, new strategies have been proposed in [118,119,120].

Detection approaches based on semantic features operate under the assumption that, while deepfake generators can synthesize low-level aspects of the signals, they are unable to replicate more intricate high-level features, and we can investigate such characteristics to discriminate real and fake data. For instance, the authors of [121] exploit classic audio features inherited from the Music Information Retrieval (MIR) community to detect speech deepfakes. Similarly, the authors of [122] leverage the lack of emotional content in synthetic voices generated via Text-to-Speech (TTS) techniques to recognize them, while those of [123] combine ASV and prosody features for the same task.

Recent trends in the speech deepfake detection field include the exploration of explainable AI (XAI) methodologies [124,125,126], the study of singing voice deepfakes [127], and the integration of pre-trained self-supervised models. These models, originally designed for Automatic Speech Recognition (ASR), are repurposed as feature extractors to create embeddings from input speech, which are subsequently used by synthetic speech detectors [128,129,130,131].

### 8.2. Video-Only Deepfake Detection

Techniques for detecting video-based deepfakes leverage visual content using a variety of approaches, including manual feature analysis and deep learning-based feature extraction.

Early forgery detection methods primarily depend on handcrafted features such as facial landmarks [27,132,133], optical flow [134], and various digital image processing techniques designed to enhance the visibility of artifacts [135]. While effective initially, these methods face limitations as deepfake generation technologies produce increasingly realistic and high-quality videos. Consequently, researchers have begun applying Deep Neural Networks (DNNs), which offer powerful feature extraction capabilities and support implicit learning of complex patterns. These methods have significantly improved the accuracy and reliability of detection processes.

As an example, the authors of [51,136] are pioneers in using DNNs to extract deep features from video frames. In [137], Convolutional Neural Networks (CNNs) and Long Short-Term Memory (LSTM) models are combined to detect fake videos generated using face-swapping techniques. The authors of [138] consider an ensemble of CNNs to detect video face manipulations, while those of [139] introduce multi-head attention and fine-grained classification to detect deepfake videos, showing that the approach is robust to low-quality videos. Liu et al. [140] analyze the frequency domain signal of deepfake videos and utilize the phase spectrum to obtain more information. Finally, the authors of [141] provide a semantic approach to deepfake detection, making use of a biological signal called photoplethysmography (PPG), an optical technique that can detect subtle changes resulting in skin color due to blood in peripheral circulation through the face.

These evolving strategies demonstrate the growing sophistication and versatility of video deepfake detection techniques, addressing both technical and physiological aspects of forgery.

### 8.3. Audio–Video Deepfake Detection

In recent years, there has been an increasing interest in the development of multimodal deepfake detection methods that can simultaneously analyze multiple modalities to achieve accurate and robust results. This comes from the fact that traditional deepfake detection methods may fall short as they often focus on either audio or video data in isolation. However, deepfakes may involve sophisticated manipulations of both audio and video streams, making them harder to detect with monomodal methods. By examining multiple modalities, multimodal detectors can identify inconsistencies or artifacts across audio and video streams, enhancing their detection capabilities. For example, a deepfake video might feature realistic facial expressions but unnatural background sounds or mismatched lip movements.

An example of such an approach is provided in [142], where the authors leverage the incongruity between emotional cues portrayed by audio and visual modalities. Conversely, the authors of [143] integrate temporal data from image sequences, audio, and video frames. Moreover, the results of [144] show that an ensemble of audio and visual baselines outperforms monomodal counterparts. The authors of [145] replace the standard MFCC features with an embedding of a deep neural-network trained for automatic speech recognition, and then incorporate mouth landmarks. In [146], the authors establish a mapping between audio and video frames by analyzing the changes in the lip opening degree, while in [32,147,148], the authenticity of a speaker is verified by detecting anomalous correspondences between their facial movements and what they say. Finally, the authors of [149] show that it is possible to perform multimodal analysis even when considering separate detectors individually trained on monomodal tasks.

Despite the great effort of the multimedia forensics community, a series of challenges remain. Concerning multimodal solutions, the need for audio–video deepfake datasets is becoming more than an urgent necessity [42,53], as the majority of current efforts have focused on developing monomodal datasets. Moreover, with the rise of large language models, there is potential to adapt similar reasoning frameworks for audio-visual analysis, which could open new avenues for research and innovation in Deepfake detection. Finally, explainability remains a significant challenge. Current methods often lack the transparency required for legal and forensic applications, making their use in courtrooms problematic. Enhancing the interpretability of these systems is essential for ensuring their reliability and acceptance in high-stakes scenarios.

## 9. Deepfakes Detection Method on Realistic Scenarios

### 9.1. Deepfake Detection of Multimedia in the Wild

The rapid advancement of deepfakes and AI-generated media has introduced significant challenges for detection systems operating in real-world environments, while Deep Learning (DL) models have demonstrated effectiveness in controlled settings, their performance often deteriorates in practical applications where uncontrolled variables, such as compression, adversarial manipulations, and evolving generative techniques, come into play [150,151]. This highlights the need for detection models that not only excel in recognizing current threats but can adapt continuously as new forms of deepfake media emerge.

A critical obstacle to achieving this lies in the tendency of DL models to become outdated when exposed to unseen data distributions, commonly referred to as data drift. The inability to generalize across evolving threats leads to gaps in detection, undermining the reliability of forensic systems. Furthermore, as models are retrained to address new threats, they risk forgetting previously learned patterns, a phenomenon known as catastrophic forgetting [152]. This creates a cycle where models must repeatedly start from scratch, limiting their long-term effectiveness.

To mitigate these issues, Continual Learning (CL) frameworks are increasingly adopted to ensure that detection models retain prior knowledge while adapting to novel manipulations [62]. CL enables systems to evolve alongside deepfake generation techniques, fostering resilience and sustained performance in diverse environments. However, even with continual adaptation, the lack of interpretability in DL models poses a barrier to their deployment in sensitive areas such as forensic analysis, legal proceedings, and media authentication. The black-box nature of these systems makes it difficult to justify or explain decisions, reducing trust and acceptance in high-stakes applications [153].

Addressing this, the integration of explainable AI (XAI) within deepfake detection pipelines is becoming a priority [154,155]. By enhancing transparency and providing insights into model behavior, XAI ensures that deepfake detection systems can be audited, understood, and trusted by non-technical stakeholders. This dual focus on adaptability and interpretability forms the foundation for next-generation detection frameworks that can operate effectively across dynamic and adversarial landscapes.

The path forward involves developing end-to-end deepfake detection systems that combine continual learning with interpretable decision-making processes. These systems must not only recognize deepfakes with high accuracy but also articulate the rationale behind their classifications, ensuring accountability and fostering confidence in their outputs. Leveraging Machine Learning Operation (MLOp) pipelines, future detection models can achieve continuous updates and seamless integration into real-world workflows, enhancing their robustness against evolving threats [156,157].

### 9.2. Deepfakes and Social Media

A highly challenging “real-world” scenario involves detecting deepfake multimedia shared on social networks [158,159,160]. To address bandwidth and storage constraints [158], social networks apply aggressive data compression and resizing. However, while these processes reduce the size of multimedia files, they also diminish the forensic features crucial for distinguishing real from fake content [158,161,162].

An initial study on GAN-generated images [159] shared via Twitter highlights the adverse effects of social network compression on deepfake detectors. Specifically, although the visual quality of shared images remained intact, the presence of forensic traces was significantly reduced. These effects were further analyzed in detail in [158].

In [160], the authors examine the challenges and advancements in media forensics as applied to social networks. The study addresses growing concerns regarding the authenticity and reliability of digital media shared on these platforms, focusing on challenges that impact source attribution algorithms [163] and multimedia verification [161]. For multimedia verification, particular attention was given to assessing whether multimedia content aligns with its descriptive text. The authors of [160] discuss emerging challenges such as the proliferation of deepfakes and the use of bots to spread disinformation. They emphasize the need for advanced forensic tools to counter these sophisticated manipulation techniques.

**Figure 6:**
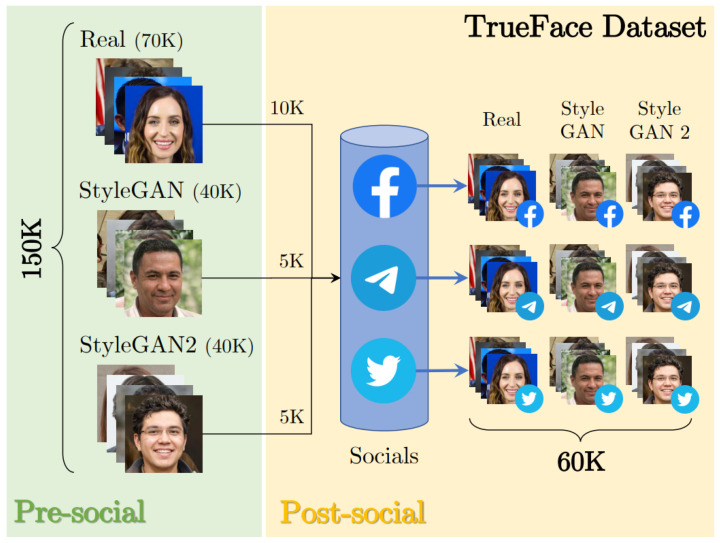
The TrueFace Dataset [158], comprising 80k fake images generated with StyleGAN models and 70k real images, of which 60k have been also shared on three distinct social networks.

In [158], the authors assembled a large and diverse dataset, visible in Figure 6, comprising 80k fake images generated with StyleGAN models and 70k real images sourced from several state-of-the-art datasets [37]. The study also provides insights into the extent and impact of compression applied by Twitter, Facebook, and Telegram. Furthermore, the authors demonstrated how this dataset could be utilized to fine-tune new detectors, preserving their accuracy on social network-compressed images while avoiding “*catastrophic forgetting loss*” [164].

Interestingly, the authors of [165] showed that while social networks degrade the forensic artifacts used by real vs. fake detectors, they introduce new traces that can help reconstruct the multimedia’s life cycle and identify the social networks where it was shared. Although the life cycle of multimedia does not directly reveal whether the content is real or fake, it aids in recovering versions closer to the original, uncompressed file, thereby enhancing detection accuracy.

In the last few years, fake images have increasingly been used on social networks to impersonate humans with realistic profiles and engage in various inauthentic activities [166], generating a growing interest in the topic and the development of methods able to detect these images.

In their work, Yang et al. [166] presented a systematic analysis of fake accounts that use GAN-generated faces on X (formerly Twitter) and a heuristic based on the eye coordinates in GAN-generated images for identifying such accounts. The authors [166] observed that GAN-generated images of faces tend to place the eyes in approximately constant coordinates. Astonishingly, the authors, following their heuristic, estimate a total of 10,000 active accounts using GAN-generated images of faces per day on X.

Similarly, Ricker et al. [167] provided a systematic, large-scale study of AI-generated profile images on X. They propose a multi-stage pipeline based on a ResNet50 [168] to distinguish real from fake images. In their study, Ricker et al. [167] estimate a total number of accounts using GAN-generated faces as profile pictures very close to the estimate provided by [166].

Maier and Riess [162] extended the studies on detecting fake GAN-generated images on social networks to include images generated by diffusion models. In their paper, the authors propose a Bayesian neural network (BNN) for detecting synthetic images. The BNN excels in identifying out-of-distribution inputs and achieves performance comparable to state-of-the-art detectors while offering more reliable predictions.

Accordingly, Kumar et al. [169] study deepfake implementation methods and their detection using GAN-based deep convolution models. They also provide a comparative analysis of the proposed GAN with other models using Inception Score (IS) and Fréchet Inception Distance (FID) metrics.

Despite recent progress in deepfake detection on social networks, the problem is far from being solved. Indeed, social network image processing algorithms are constantly updated, and fake image detectors, trained on real and fake images shared on social networks during the last few years, need to be fine-tuned according to these updates [158].

To make things worse, Lago et al. [170] showed how fake images are becoming increasingly indistinguishable to humans from real images.

This makes it necessary to develop methods that are constantly updated through continual- and few-shot learning [62,171].

Finally, preliminary studies on videos shared on social networks [172] reveal similar effects to those observed with images. One such study [21,172] investigated the impact of social network compression on FaceForensics [172] videos shared on Facebook and YouTube. The findings align with those for images [172] and introduce a new dataset of shared videos for fine-tuning real vs. fake detectors.

### 9.3. Detection of Deepfake Images and Videos in Adversarial Setting

An additional problem affecting virtually all the deepfake forensic techniques developed so far is that such techniques are thought to operate in a benign setting, that is, by neglecting the possible efforts made by an adversary to mislead the forensic analysis. Yet, recent studies [173,174] have shown how easy is to generate adversarial contents capable of deceiving image and video processing techniques based on DL, when the adversary is informed about the details of the tools employed by the analyst. Some works [175,176] have also studied the transferability of adversarial examples to networks different than those targeted by the attack, opening the way to the development of powerful attacks even when the attacker is unaware, or only partially aware, of the techniques used by the analyst. This includes recent techniques based on Contrastive Language-Image Pretraining (CLIP) features employing a vision transformers as backbone. In [177], for instance, it is shown that methods based on CNNs and CLIP features share the same vulnerabilities against adversarial attacks, even if the kind of perturbations introduced within the attacked images in the two cases is quite different. For this reason, attacks against CNN-based techniques usually do not transfer to methods based on vision transformers and CLIP features, and vice versa.

Often, it is not even necessary that the adversary applies sophisticated attacks relying on the full or partial knowledge of the to-be-attacked system. By relying on the lack of robustness and the generalization capabilities of the forensic tools outlined in Section 9.1, the adversary may simply process the deepfake content in such way to prevent a correct forensic analysis, or at least degrade its performance to a point that makes it unusable. Some examples of this kind of attack, often referred to as *laundering attacks*, include the application of moderate to strong lossy compression, geometric processing of images and videos, noise addition, histogram stretching, and many others.

Understanding and ensuring the security of deepfake forensic tools is a crucial problem if such tools have to be used under the intrinsically adversarial conditions typical of multimedia forensics applications. For this reason, several efforts have been made to defend against adversarial attacks [178,179], both in the realm of computer vision applications and multimedia forensics. Still, no general effective solutions have been found yet [180]. Among the solutions developed so far, adversarial training [181] has received some consensus and has proven to at least mitigate the effectiveness of adversarial attacks in computer vision applications. As argued in [182], adversarial training forces DL models to focus on robust, possibly semantic, features, which are inherently more difficult to attack. Whether such a beneficial effect of adversarial training also applies to deepfake forensic applications is still an open problem. It is not clear, in fact, if in multimedia forensics the equivalent of semantic computer vision features exist.

With regard to laundering attacks, the solutions proposed so far are similar to those already discussed in Section 9.1, given that, ultimately, the effectiveness of laundering attacks can be drastically reduced by improving the robustness and generalization capabilities of the forensic tools. A common approach to do so involves the use of data augmentation techniques that enrich the training set with processed samples, thus improving the robustness against the processing operators used for data augmentation. Yet, accounting for *all* possible kinds of processing during training is clearly unfeasible. Among the solutions proposed so far, one is the possibility of identifying a kind of *worst possible laundering attack*, an approach we have included in the training procedure. An example of such an approach is the inclusion within the data augmentation pool of a Print and Scan (P&S) simulator [183]. The P&S process, in fact, introduces various distortions that tend to eliminate or significantly diminish the subtle statistical traces often relied upon by synthetic image detectors. In [183], the authors contend that training a detector to maintain its performance on images that have undergone P&S compels it to depend on robust features that remain discernible even after substantial transformations. Given the impracticality of creating a large dataset of physical P&S images, in [183] the authors utilize a CycleGAN to simulate the P&S process. The CycleGAN P&S simulator was initially trained on image patches (patch P&S) and then fine-tuned on full face images (full P&S). This approach was tested on a synthetic image detection task, using both common processing and P&S simulation in the augmentation list. Validation was carried out on natural image datasets like CelebA-HQ and FFHQ (N), alongside synthetic datasets generated by StyleGAN2 (SG2), Latent Diffusion (LT), and Taming Transformers (TTs). Experimental results, summarized in Table 2, demonstrate that detectors trained with P&S-augmentation achieve greater robustness to unseen processing techniques, including JPEG2000 compression, WebP, filtering, and color transformations, without sacrificing performance on the processing tasks used during augmentation.

Another example of the worst case attack approach is given in [184], where a double JPEG detector is trained on a selected pool of images subject to a set of worst case laundering attacks belonging to different processing categories.

Despite all the efforts made, even for laundering attacks, a definitive solution has not been devised yet, thus adding yet another point to the *to-do* list of multimedia forensic researchers.

## 10. Active Authentication

### 10.1. Active Deepfake Detection

Deepfake detection has been approached so far by means of passive techniques [185,186,187] that operate *after the fact*, meaning they are applied after the forged content has been created, distributed, and potentially manipulated. Conversely, active methods work proactively by pre-processing media and embedding a fingerprint to facilitate subsequent analysis. This is obviously an important change in the paradigm and it foresees the capacity to intervene at the stage of the content generation process and, above all, the active cooperation of the entity that trained the media-generation network. This last option is not so easy to achieve but it can be motivated by the fact that entities and organizations that have spent effort in training models for image/video generation should be willing to protect them. Moreover, they should also have an interest in tracking the multimedia content they are going to produce through the interactive services they make available to the common end-users. In addition, there will also be the need to understand if such fingerprinted contents are used as a basis to construct new trained models for deepfake creation. So it could be crucial to individuate if a particular deepfake document (e.g., an image) has been produced by a specific generation system in order to perform source attribution and authenticity assessment. One of the basic ideas behind active fingerprinting for active deepfake detection was presented in [100]. The method can be divided into two main steps. The first is to train a model, constituted by a couple encoder–decoder (Enc/Dec), that, given an image *I* as input, is able to embed a watermark *w* (a string of *n* bits) into a reconstructed image $I^{\prime }$. The training process has to minimize a loss function composed of two terms, a mean squared error between the input image and the watermarked-reconstructed one ($L_{MSE}$) and a binary cross entropy ($L_{BCE}$) between the input watermark *w* and the decoded one $w^{\prime }$. When this network is trained, it is adopted for the second step, which is the actual active fingerprinting. The encoder (Enc) is now used to embed a specific watermark $w_{i}$ in a dataset of images so to obtain a watermarked dataset that contains images marked with $w_{i}$. Such a dataset is then used to train a certain generative network ($GAN_{i}$ composed of a generator $Gen_{i}$ and a discriminator $Dis_{i}$) that will learn to generate images with the watermark $w_{i}$ embedded. After that, if such trained image generator $Gen_{i}$ will be used to create a deepfake content (e.g., a deepfake image), it will be possible, by taking the decoder Dec, to extract the watermark $w_{i}$ from such image in order to check its provenance and, at the same time, to determine that the content is not pristine. The same process can be extended to *N* different kinds of generation systems (1≤i≤N) to perform fingerprinting of the diverse deepfake generative models. Obviously, these kinds of approaches need to be tested and improved to satisfy diverse requirements to become actually efficient; in fact, it is fundamental to balance the need to grant robustness against possible image processing with the request to preserve image quality and to provide a sufficient capacity in information hiding. All this opens the way to possible new research opportunities in the field of active deepfake detection.

### 10.2. Efficient Media Origin Authentication

Customary deepfake detection methods, both passive and active, are subject to false positives and false negatives, whose rate highly depends on the employed method and the goodness of the training data. False positives are due to various factors, such as the complexity of the content, the quality of the training data, or the intrinsic limitations of the detection algorithm itself. False negatives could happen if the deepfake is very well made, or in general if the detection method fails to recognize certain patterns or features that indicate a deepfake. On the other hand, cryptographic signatures are “almost perfect” from this point of view, in the sense that false negatives (i.e., authentic signatures which are not recognized so) are zero and false positives (i.e., fake signatures that are taken as authentic) are considered computationally infeasible to forge. This suggests that cryptographic signatures could be used fruitfully to detect deepfakes (or better, the absence of deepfakes) with perfect precision. In this direction, the work-in-progress standard JPEG Trust [188] by the Joint Photographic Experts Group (JPEG) aims to establish trust in digital media by addressing aspects of authenticity, provenance, and integrity. The JPEG Trust will provide a framework for establishing trust in media through secure annotation of media assets throughout their life cycle, using cryptography as a key component. Cryptographically signing media allows deepfakes to be repudiated by the interested person, since signatures will be absent or invalid. Such an anti-fake signature should allow “good” manipulation of the original file (at least cropping) and disallow “bad” manipulation, but also be space efficient to save bandwidth on web servers once the media file is disseminated. Unfortunately, customary signature schemes like ECDSA do not have these properties.

To address this challenge, a solution could rely on novel aggregatable signatures, such as the Boneh–Lynn–Shacham (BLS) signature [189,190], which has been successfully used in blockchain technologies like Ethereum 2.0 to optimize storage (https://github.com/ethereum/consensus-specs/blob/dev/specs/phase0/beacon-chain.md#bls-signatures, accessed on 15 February 2025). The BLS signature scheme makes use of a novel form of cryptography called pairing-based cryptography, which allows for a plethora of new functionalities like attribute-based encryption [191,192,193]. Aggregatable signatures could be employed in JPEG (possibly within the JPEG Trust standard itself) in such a way to permit benign alterations of the image like cropping while preventing malicious tampering, without increasing too much the bandwidth occupation on web servers.

The system is composed of three entities: Signer, Publisher, and Verifier. The Signer represents the interested person (or an agency that manages their public reputation), which is the only entity in possession of the private key. The Signer will sign the individual JPEG blocks that make up the image, and the signatures produced will be stored as metadata within the JPEG file. The JPEG file, along with the related BLS signatures, will then be passed to the Publisher, which represents a news agency interested in publishing photographic material through the web. The Publisher can possibly crop the original image, by removing the JPEG blocks and the related signatures that are outside a given cropping rectangle. The remaining signatures (those relative to the blocks inside the cropping rectangle) can be aggregated into a single signature by the Publisher, leveraging the aggregability properties of BLS. This allows the Publisher to drastically reduce the size of the JPEG file, therefore saving a lot of bandwidth on the web server. Finally, the Verifier represents the final user, which wants to verify the origin of the (possibly cropped) image. The Verifier extracts the aggregated signature embedded in the JPEG file downloaded from the Publisher’s web site and verifies it by means of the Signer’s public key.

### 10.3. Trusted Remote Media Processing on Cloud and Edge Computing Systems

In the analysis of new techniques and future challenges, it is important to mention the new use of Distributed Systems in the context of AI, one of the main modern outcomes of which is the creation of the *Transformer* paradigm [194]. Here, OpenAI has also benefited from this, having leveraged the power of the cloud in conjunction with Big data in creating ChatGPT, based initially on GPT-3 then on the GPT-4 architecture [195,196].

In this work, we focus on emerging media production contexts, i.e., systems based on the IoT (Internet of Things), which is playing an important role increating the Digital Smart Data Cities of the near future. Here, citizens can interact with the environment and benefit from a plethora of advanced services, such as video surveillance, intelligent traffic lightning, air quality detection, and new advanced fire and flooding management systems. From a technological point of view, sensors and actuators able to automate new services are strategic in their configuration, and optimization, hence, even more digital infrastructures need to adapt their behavior to the specific needs of the context; this represents a big challenge both in terms of system design and security. Deepfake media detection in these scenarios represents a challenge due to the nature of possible manipulations of future digital content in citizens’ daily lives; hence, a more holistic approach should be considered where media production happens, since Edge Computing Systems [197] need to be taken into account. In just 20 years, with the objective of increasing system response and reduce communication latency, computation moved from mainframes and computing rooms towards Cloud Computing, Fog Computing, and lastly, Edge Computing. A Federated Cloud–Edge infrastructure is considered, where different administrative domains are in place and where Artificial Intelligence and Machine Learning software artifacts, in the assertion of Federated Learning even at the Edge, help to distribute intelligence in this scenario.

Multimedia acquisition devices based on the IoT generate an unprecedented amount of data, leading to more frequent instances of manipulated and fake data [198], hence, the need to develop cloud-based video big data analytics frameworks. A distributed approach in video recording and elaboration systems, such as video surveillance systems based on IP cameras, is highly recommended to overcome the maximum storage or throughput limitation of network video rRecorders installed on single machines. To perform such a variety of tasks, and to be able to modify a device’s behavior on-demand, the Function as a Service (FaaS) computational paradigm has generally been adopted. FaaS allows several minimal applications to be defined and one or more instances of these applications to be run on the same device at the same time. The FaaS framework relies on two configuration approaches: a local configuration file, generally YAML, or a secure remote server. However, both come with limitations: a local file configuration requires direct access to the device, physically or through a secure connection, to modify it; while, although a remote server can store and send updated configuration files, it might be vulnerable to well-known cyberattacks suck as Man-in-the-middle (MITM) or Distributed Denial of Service (DDoS), making it unusable and unreliable. To overcome such limitations, it is possible to benefit from three technologies that have been increasingly recognized to be able to address information access problems and system trustworthiness in different application domain: Federated Learning [199], the Blockchain, and the IPFS (InterPlanetary File System) starting at the Edge:

- Federated Learning is a decentralized approach to training machine learning models. In traditional machine learning, data are centralized in the Cloud, where a single model is trained on the entire dataset. Federated Learning, conversely, allows the training of machine learning models across multiple decentralized devices or servers that hold local data samples without exchanging them. Moreover, Federated Learning at the Edge refers to the application of Federated Learning techniques on Edge devices, such as IoT devices, or Edge Servers. This approach combines the benefits of Federated Learning, which ensures data privacy and reduces communication costs, with the advantages of Edge Computing Systems, which enables data processing and model training to occur closer to where the data are generated, hence, fake media might not exit from the Edge. New approaches can be adopted like the Federated Learning system deployed into Web Browsers for enlarging the the Cloud–Edge–Client Continuum capabilities [200,201].
- The use of the Blockchain, supported by the flexibility and robustness of smart contracts, allows the combination of the well-known FaaS paradigm with the intrinsic features of data non-repudiation and immutability, replacing the service configuration with a smart contract, guaranteeing protection against distributed cyber-attacks [202].
- The IPFS is a distributed system for storing and accessing files. Since the block size of the Blockchain does not allow storing files, these can be uploaded to this special file storage, which produces a unique hash value to be used as a key to access its content [203].

## 11. Discussion

The advances and challenges discussed in this paper highlight the evolving landscape of deepfake detection, authentication, and forensic analysis. As synthetic media generation continues to improve, detection methodologies must adapt to maintain their effectiveness in real-world scenarios. This section, devoted to a generic discussion, summarizes the key points presented in the paper, evaluating current detection strategies, identifying existing limitations, and outlining future research directions to improve the robustness, efficiency, and ethical use of deepfake detection technologies.

### 11.1. Evaluating Deepfake Detection Strategies: Challenges, Advances, and Future Directions

The findings and methodologies presented in this comprehensive review highlight the dynamic interplay between the rapid advancements in deepfake generation technologies and the equally rapidly evolving detection strategies. Table 3 provides a structured comparison of detection methods analyzed in this paper, outlining their advantages and weaknesses while referencing the sections where they are examined in detail. As manipulation techniques become increasingly sophisticated, detection methods must continually adapt to address new forensic challenges, each requiring different approaches and levels of robustness. In the short term, one of the most pressing challenges remains the availability of diverse and high-quality datasets for training and evaluating deepfake detection models. Existing datasets often fail to represent the full spectrum of real-world manipulations, limiting the generalization capabilities of forensic models. A possible solution could be expanding dataset diversity to include a broader range of manipulations and real-world distortions. However, this approach alone is insufficient, as new deepfake techniques constantly emerge, making it impractical to cover all possible variations. Instead, future efforts should prioritize adaptive strategies, such as synthetic augmentation, domain adaptation, and meta-learning, to improve the generalization capabilities of detection models beyond their training distributions.

A key mid-term challenge is improving the adaptability and resilience of detection models, while forensic-based techniques leveraging frequency-domain analysis and spatio-temporal coherence can detect subtle artifacts, their reliance on high-quality input data and substantial computational resources limits their practicality in real-world applications. Machine learning-based approaches, particularly deep learning methods, have demonstrated strong performance on benchmark datasets, but they remain vulnerable to adversarial manipulations and suffer from high computational costs. Another major limitation across detection strategies is their sensitivity to media compression. Social media platforms, where deepfake content is frequently disseminated, apply aggressive compression that often degrades forensic traces. Future research should focus on developing robust detection frameworks that integrate compression resilience without compromising detection accuracy. Continual learning frameworks present a promising solution, allowing models to evolve alongside new threats while mitigating catastrophic forgetting. However, the lack of universally accepted benchmarks for evaluating these methods remains a significant bottleneck, hindering their large-scale deployment. Additionally, the integration of explainable AI into forensic pipelines is becoming increasingly important, providing interpretability to detection decisions and improving their usability in real-world forensic investigations. Future works should balance model transparency with detection performance, ensuring that forensic tools are both interpretable and robust.

In the long term, an effective deepfake mitigation strategy must extend beyond detection models and incorporate proactive defense mechanisms such as active authentication. Embedding traceable fingerprints at the content creation stage provides a safeguard against manipulation, but its efficacy is dependent on widespread adoption by content platforms and resilience against tampering. Furthermore, regulatory frameworks must evolve to address deepfake forensics’ ethical and legal implications, ensuring a balance between content authenticity verification and fundamental rights such as privacy and freedom of expression. Given the rapid advancements in generative AI, the long-term success of deepfake detection will require a coordinated effort between researchers, policymakers, and industry stakeholders to develop scalable and adaptive solutions that align with both technological advancements and societal needs.

### 11.2. Ff4ll’S Goals in Deepfake Detection and Media Authentication

The FF4LL project is designed to address the evolving challenges of deepfake detection and media authenticity by providing comprehensive solutions that integrate detection, attribution, and authentication strategies. Given the increasing sophistication of generative models and their potential for abuse, FF4LL aims to bridge the gap between cutting-edge research and practical dissemination, ensuring that forensic tools remain robust and available in different real-world applications.

The overall goals of FF4LL can be categorized into three main points:

- **Advancing deepfake detection through multimodal approaches:** The project aims to develop robust and scalable detection frameworks capable of countering image- and audio-based forgeries. Leveraging machine learning, frequency analysis, and adversarial robustness techniques, FF4LL aims to improve detection accuracy while reducing computational overhead by addressing key limitations in this field;
- **Ensuring media authenticity through active and passive authentication:** FF4LL emphasizes the integration of proactive authentication methods to incorporate traceable fingerprints at the time of content creation. This approach, combined with device authentication and secure cloud-based media processing, ensures that the integrity of content can be verified even before forensic analysis is required, enhancing trust in digital media ecosystems;
- **Impact on cybersecurity, digital communication, and public trust:** The implications of deepfake proliferation go beyond media forensics to cybersecurity, disinformation control, and public perception of digital content. FF4LL’s contributions to secure authentication and forensic analysis aim to mitigate fraud, identity theft, and disinformation campaigns by providing governments, journalists, and digital platforms with the tools to safeguard digital communication channels.

By integrating these approaches, FF4LL provides a framework that aligns with both current challenges and future advances in synthetic media generation. The project’s interdisciplinary collaboration among AI researchers, forensic experts, and many others ensures that deepfake detection and authentication technologies evolve in parallel with emerging threats, promoting a more secure and trusted digital landscape.

### 11.3. Ethical Concerns and Governance in Deepfake Detection

Deepfake detection technology, while critical to safeguarding the integrity of digital media, raises significant ethical issues that require careful consideration. One of the most pressing issues is privacy. The effectiveness of these tools relies on the analysis of facial features, voice patterns, and other biometric data, which are inherently sensitive. The collection and storage of this information presents substantial privacy risks, especially in the event of unauthorized access. These data, if misused, could lead to identity theft, surveillance, or a serious breach of personal privacy. Therefore, while the intent of using deepfake detection technology may be to protect people from deception, it also exposes them to potential violations of their biometric security. Beyond privacy, the issue of disinformation poses a significant ethical dilemma. Deepfakes have already demonstrated their ability to manipulate public opinion by creating realistic but false narratives. Even with advances in detection technologies, the damage caused by misleading content can be rapid and widespread before it is actually exposed. Moreover, a paradox emerges: the evolution of detection tools drives the development of more sophisticated deepfakes. Malicious actors, learning from detection algorithms, can create increasingly deceptive content. This not only reduces public trust in digital media but also compromises the entire information system. To address these ethical challenges, it is essential to develop comprehensive standards and policies that govern the use of deepfake detection technology. A primary measure is the implementation of strict data privacy standards. These standards should ensure that biometric data are securely stored, anonymized, and used only for detection purposes. Compliance with international privacy standards, such as the General Data Protection Regulation (GDPR) or the California Consumer Privacy Act (CCPA), is critical to maintain transparency and protect users’ rights. It is also necessary to implement robust consent mechanisms that allow individuals to control their biometric data, including the right to request deletion. In terms of privacy protection, it is essential to ensure transparency and accountability to reduce the risks associated with deepfake detection technology. It is important that deepfake content is clearly labeled and detection tools are used so that users know whether a piece of media is authentic or not. In addition, the creation of independent institutions to monitor these technologies would help ensure fairness and prevent abuse. Thus, although deepfake detection technology plays a key role in countering digital deception, its ethical implications must be carefully managed. Privacy concerns, the risk of misinformation, and the potential for misuse underscore the need for balanced and comprehensive policies. By implementing strict data privacy standards and promoting transparency and responsibility, society can take advantage of the benefits of deepfake detection technology while minimizing ethical risks. This balanced approach will be critical to safeguarding both individual rights and the integrity of digital information.

To counter the disinformation risks posed by deepfakes, FF4LL researchers focus on robust detection strategies that remain effective even when synthetic content is highly compressed and manipulated for adversarial purposes. These techniques reduce the rapid dissemination of false information before it can significantly influence public opinion. In addition, FF4LL studies explainable AI (XAI) approaches to improve the interpretability and forensic reliability of deepfake detection, ensuring that decisions are transparent and defensible in legal and cybersecurity contexts. By balancing technological advances with privacy safeguards, transparency, and ethical considerations, FF4LL aims to strengthen public trust in digital media while minimizing the ethical risks associated with deepfake detection. Through the collaboration of AI experts, forensic and legal experts, the project is helping to create a safer, more responsible and ethical media landscape.

## 12. Conclusions

This study presents a comprehensive analysis of the current landscape of deepfake media forensics, focusing on detection, attribution, and authentication techniques. The work highlights the critical challenges posed by synthetic media in controlled and real-world scenarios, as well as the technological advancements developed to counteract these threats. While significant progress has been made, the field faces persistent challenges related to scalability, real-time applicability, and robustness in dynamic environments. Scalability remains a pressing issue, as many current state-of-the-art techniques require high computational resources and extensive labeled datasets, which limit their adoption in large-scale or resource-constrained settings. Addressing this challenge requires the development of lightweight, energy-efficient algorithms, potentially leveraging model pruning, quantization, or distillation to maintain performance while reducing computational demands. Real-time applicability is another critical hurdle. Many detection algorithms, particularly those employing deep learning, suffer from latency issues, making them unsuitable for applications such as live media verification or streaming platforms. To overcome this limitation, future research should focus on optimizing inference times by using edge computing frameworks and designing architectures tailored for low-latency environments, such as transformer-based models adapted for efficiency. The robustness of detection systems in dynamic environments is equally crucial. As generative technologies evolve, new types of deepfake manipulations constantly emerge, challenging the adaptability of current detectors. Adversarial attacks, in particular, pose a significant threat, as they can exploit weaknesses in detection systems to evade scrutiny. Enhancing the generalization capabilities of detection models is vital and can be achieved through continual learning frameworks, adversarial training, and extensive data augmentation strategies that simulate diverse real-world conditions. These approaches can help models retain knowledge of previously encountered manipulations while adapting to novel threats. Beyond technical advancements, addressing ethical considerations is essential to ensure the responsible use of these technologies. Standardized guidelines should be established to tackle privacy concerns, prevent misuse, and promote transparency in the development and application of detection tools. Prioritizing initiatives like explainable AI (XAI) can enhance the interpretability and trustworthiness of detection systems, particularly in forensic and legal contexts where their outputs may significantly influence critical decisions. In conclusion, while the advancements in deepfake media forensics are commendable, a concerted focus on technical innovation and the establishment of robust ethical frameworks is essential to address the challenges posed by synthetic media and ensure the integrity and authenticity of digital content in an increasingly complex technological landscape.

## Figures and Tables

**Table 1:** Comparison of topics in deepfake research, highlighting their focus, techniques, proactivity, practical applications, and constraints addressed.

Aspect	Deepfake Detection (Section 3, Section 5 and Section 6)	Attribution and Recognition (Section 7)	Passive Methods (Section 8)	Realistic Scenarios (Section 9)	Active Authentication (Section 10)
**Focus**	Detecting fake media	Identifying creators/tools	Forensic analysis of content	Detection methods in real-world contexts	Embedding authenticity proactively
**Techniques**	ML, DL, handcrafted features	Source tracing, watermarking	Artifact analysis, temporal signals	Compression-aware, robustness frameworks	Watermarking, blockchain, cryptography
**Proactivity**	Reactive	Reactive	Reactive	Context-adaptive	Proactive
**Practical Use Case**	Identifying deepfakes	Tracing fake origins	Low-resource environments	Addressing low-quality, compressed content	Ensuring authenticity before distribution
**Constraints Addressed**	Feature engineering, ML/DL gaps	Accountability	Lack of active authentication	Real-world deployment challenges	Pre-distribution authenticity assurance

**Table 2:** Accuracy of synthetic image detectors when faced with image processing operations that were not part of the augmentation set, comprising JPEG2000 and WebP compression, Median (Med), Gaussian (GF), and Wiener (WF) filtering, brightness and contrast transformations (CT), hue modification in the HSV space (HSV), and P&S simulation trained on patches (P&S P).

Task	Model	Augment	JPEG 2000	WebP	Med	GF	WF	CT	HSV	P&S P
N vs. SG2	XNet	Conventional	0.87	0.72	0.82	0.91	0.56	0.98	0.86	0.50
		+ patch P&S	0.93	0.92	0.93	0.94	0.90	0.98	0.93	0.81
		+ full P&S	0.94	0.84	0.86	0.97	0.92	0.97	0.91	0.50
	ResNet	Conventional	0.94	0.92	0.91	0.96	0.96	0.97	0.93	0.95
		+ patch P&S	0.96	0.96	0.95	0.97	0.96	0.97	0.93	0.97
		+ full P&S	0.95	0.95	0.92	0.96	0.95	0.96	0.94	0.96
N vs. DM	XNet	Conventional	0.58	0.67	0.69	0.98	0.54	0.99	0.99	0.50
		+ patch P&S	0.77	0.95	0.78	0.84	0.78	0.99	0.99	0.89
		+ full P&S	0.63	0.93	0.75	0.86	0.59	0.99	0.98	0.50
N vs. TT	XNet	Conventional	0.91	0.96	0.86	1.0	0.91	1.0	0.99	0.5
		+ patch P&S	0.99	0.99	0.96	0.99	0.99	0.99	0.99	0.97
		+ full P&S	0.94	0.99	0.90	0.99	0.98	0.99	0.99	0.50

**Table 3:** Comparison of deepfake detection methods discussed in this paper, along with their strengths, weaknesses and the Sections where they are examined.

Method	Description	Strengths	Weaknesses	Relevant Sections
Forensic-Based Detection	Uses handcrafted features to detect anomalies in digital content (e.g., artifacts, lighting inconsistencies).	Interpretable; Can detect handcrafted manipulations; Computationally efficient.	Struggles with advanced AI-generated content; Sensitive to compression artifacts.	Section 3.2, Section 3.4, Section 8 and Section 9
Machine Learning-Based Detection	Employs deep learning models like CNNs and Transformers to learn patterns from large datasets.	High accuracy on benchmark datasets; Can generalize well if trained on diverse data.	Requires large labeled datasets; Prone to adversarial attacks and dataset bias.	Section 3.1, Section 8 and Section 9
Biometric-Based Detection	Leverages physiological and behavioral cues (e.g., blinking, heart rate, gaze tracking) to detect manipulations.	Difficult for deepfake models to replicate; Based on real human characteristics.	Requires high-resolution input; Can fail under occlusions or low-light conditions.	Section 3.3
Continual Learning for Deepfake Detection	Enables models to continuously adapt to new types of deepfake manipulations over time.	Improves model adaptability; Reduces catastrophic forgetting; Enables learning from evolving deepfake techniques.	Requires large-scale continual training; Susceptible to domain shifts; High computational cost.	Section 5 and Section 9
Deepfake Attribution and Model Fingerprinting	Uses techniques to analyze residual patterns in synthetic media to determine the source generative model.	Traces deepfake origins; Identifies generative model fingerprints; Useful for forensic tracking.	Limited effectiveness if the attacker modifies or fine-tunes the generator; Relies heavily on dataset biases.	Section 7
Methods for Explainability in Deepfake Detection	Focuses on making deepfake detection models more interpretable and explainable to improve forensic reliability.	Enhances trust in detection models; Provides interpretability for forensic investigations; Supports model debugging.	No standard framework for explainability; May reduce model accuracy due to interpretability constraints; Black-box AI models remain dominant.	Section 6 and Section 9
Active Authentication	Uses proactive security mechanisms such as watermarking and cryptographic signatures to ensure authenticity.	Provides proactive protection; Can grant deepfake detection and generation source attribution	Depends on adoption by platforms; Can be circumvented if attackers remove metadata.	Section 10

## Data Availability

Data are contained within the article.

## Abbreviations

The following abbreviations are used in this manuscript:

FaaS	Function as a Service
MITM	Man-in-the-Middle
DDoS	Distributed Denial of Service
XAI	Explainable Artificial Intelligence
GANs	Generative Adversarial Networks
DMs	Diffusion Models
SOTA	State of the art
